# Associations among IGF-1, IGF2, IGF-1R, IGF-2R, IGFBP-3, insulin genetic polymorphisms and central precocious puberty in girls

**DOI:** 10.1186/s12902-018-0271-1

**Published:** 2018-09-19

**Authors:** Hua-Pin Chang, Shun-Fa Yang, Shu-Li Wang, Pen-Hua Su

**Affiliations:** 1 Department of Nursing, Asia University, Taichung, Taiwan; 2 Department of Nursing, Asia University Hospital, Taichung, Taiwan; 3 Institute of Medicine, Chung Shan Medical University, Taichung, Taiwan; 4 Department of Medical Research, Chung Shan Medical University Hospital, Taichung, Taiwan; 5 National Institute of Environmental Health Sciences, Zhuman, Taiwan; 6 The Department of Public Health, China Medical University, Taichung, Taiwan; 7 Department of Pediatrics, Chung Shan Medical University Hospital, Taichung, Taiwan; 8 School of Medicine, Chung Shan Medical University, Number 110, Section 1, Chien-Kou North Road, Taichung, 402 Taiwan

**Keywords:** Insulin-like growth factor 1, Insulin-like growth factor 2, Central precocious puberty, Single nucleotide polymorphism, Insulin-like growth factor binding protein 3, Insulin-like growth factor receptor

## Abstract

**Background:**

Insulin and insulin-like growth factor (IGF)-1 coupled with growth hormone helps control timing of sexual maturation. Mutations and variants in multiple genes are associated with development or reduced risk of central precocious puberty (CPP).

**Methods:**

We assessed single nucleotide polymorphisms (SNPs) in the *IGF-1, IGF-2, IGF-3*, IGF-1 receptor (*IGF1R*), IGF-2 receptor (*IGF2R*), and IGF -binding protein 3 (*IGFBP-3*) genes, and their association with demographics and metabolic proteins in girls with CPP. Z-scores of height, weight, and body mass index (BMI) were calculated with the WHO reference growth standards for children.

**Results:**

IGF-1 serum levels of CPP group exhibited a higher correlation with bone age, z-scores of height and weight, and luteinizing hormone (LH) than those of control group, regardless of BMI adjustment. In the CPP group, height was associated with *IGF-2(3580)*, an adenine to guanine (A/G) SNP at position + 3580. BMI in the CPP group was associated with *IGF-2(3580), IGF1R*, and the combinations of [*IGF-2(3580) + IGF2R*], and [*IGF-2(3580) + IGFBP-3*]. Body weight in the CPP group was associated with the combination of [*IGF-2(3580) + IGFBP-3*] (*p* = 0.024). Weight and BMI were significantly associated with the combination of [*IGF-2(3580) + IGF2R + IGFBP-3*] in the CPP group. These associations were not significantly associated with z-scores of weight, height, or BMI. The distribution of these genotypes, haplotypes, and allele frequencies were similar between control and CPP groups.

**Conclusions:**

These known SNPs of these IGF-1 axis genes appear to play minor roles in the risk for development of CPP.

**Electronic supplementary material:**

The online version of this article (10.1186/s12902-018-0271-1) contains supplementary material, which is available to authorized users.

## Background

Precocious puberty (PP) is defined as early development of puberty in females and males (before 8 years and 9 years of age, respectively). The two types of PP, central precocious puberty (CPP) and pseudo or peripheral precocious puberty (PPP) differ in their etiology. Secondary sex characteristics of PPP develop early due to excessive hormonal secretion from reproductive tumors or adrenal hyperplasia, and PPP is considered gonadotropin-independent. In contrast, CPP is gonadotropin-dependent and involves the premature maturation of the hypothalamic-pituitary-gonadal axis which induces the early secretion of testosterone from boys’ testes and estrogens from girls’ ovaries. Although the cause of CPP is not elucidated in most cases, CPP can be associated with obesity, brain structural abnormalities, head injuries, and products that contain compounds which mimic hormones including cosmetic products, some insecticides, and some foods [1]. CPP causes early onset of menarche and initial breast development in girls, psychosocial challenges, and early epiphyseal fusion in bones which reduces further growth spurts and thus may lower final height. Treatment has the goal of preserving growth and height potential and can involve gonadotropin-releasing hormone (GnRH) analogs [2]. Treatment for CPP did not significantly affect the risk of cancer death, obesity, or metabolic disorders in 30- to 50-year-old women who had had CPP [3].

Environmental and metabolic factors may influence the initiation of puberty in 20 to 50% of cases [4]. Poor nutrition may delay puberty while obesity promotes earlier initiation of puberty in girls [5–9].

The hormones, insulin, insulin-like growth factor-1 (IGF-1), and growth hormone (GH) are linked to precocious puberty [10–12]. GH and the IGF signaling pathways play major roles in regulating endocrine secretions involved in growth and sex maturation. The complex IGF network involves several growth factors (IGF-1, IGF-2), high affinity insulin-like growth factor binding proteins (IGFBP3, IGFALS) and cell surface receptors (IGF-1R). Girls with CPP have higher insulin and IGF-1 blood levels than girls without PP [11–13]. In animal studies, insulin or IGF-1 stimulation augments GnRH secretion, confirming that insulin receptors (IRs) and IGF-IR are present on GnRH neurons. Furthermore, puberty is delayed in female mice lacking IGF-1R, but not in males [14], suggesting a role of IGF-1 signaling in timing of puberty in females.

Mutations in the *MKRN3* gene and the *KISS* gene are highly associated with CPP [15, 16]. However, the influence of the IGF axis genes on puberty in humans, especially CPP, is unclear. As tangential evidence, girls diagnosed with PP have a higher risk for developing breast cancer than girls without PP [17]. Abnormalities in the IGF signaling pathways affect progression of breast cancer [18].

The hypothesis of this study is that polymorphisms in one or more of the IGF genes may influence sex hormonal changes and affect the development of precocious puberty. Since members of the IGF family are involved in the onset of puberty, we aimed to identify polymorphisms in insulin (*INS*), *IGF-1*, *IGF-2*, IGF-1 receptor (*IGF1R*), *IGR2R*, and IGF binding protein 3 (*IGFBP-3*) that may alter the risk for development of central precocious puberty.

## Methods

### Study population

This two-cohort study assessed ≤8 year old girls who showed development of secondary sex characteristics and sought care in the genetic/metabolic outpatient department at the Hospital of Chung Shan Medical University. The study included a total of 489 girls with 264 girls in the CPP group, and 225 girls in the early puberty (EP) control group. During the study’s implementation period, all girls received treatment and consultation. All girls were examined for bone age (BA), weight, and height; their blood samples were collected for estradiol (E2), luteinizing hormone (LH), and luteinizing hormone-releasing hormone (LHRH) analysis; and their BA/CA (bone age/chronological age) ratio and BMI were calculated during their first visit. Follow-up visits occurred every 3 months.

Girls were diagnosed with CPP if the girls’ bone age examination results were greater than their age growth, estradiol was ≥10 pg/ml, and highest LHRH and LH values were ≥ 10 ml U/ml [19]. Girls with CPP received consultation, health education, and treatment from a genetic/metabolic counselor. Eligibility criteria for the CPP group included continuing outpatient treatment at this hospital after outpatient examination and diagnosis with CPP.

Exclusion criteria for the CPP group consisted of refusal to sign a consent form or having a disease that may have caused CPP, such as a chromosome anomaly; a noncancerous tumor in the brain or pituitary gland, brain injury; an infection in the brain (e.g. meningitis); radiation or chemotherapy for cancer treatment.

If the girls’ bone age, estradiol, LH, and LHRH test results did not fully meet the diagnostic standard in the first visit and they only had emergent secondary sex characteristics, they were considered to have EP. The girls with EP received consultation and health education from a genetic/metabolic counselor and continued to receive outpatient follow-up. Exclusion criteria for the EP group were a GnRH homolog treatment, failure to continue to receive follow-ups, refusal of family members to sign the participation consent forms, or the aforementioned diseases or treatments in the exclusion criteria for CPP.

### Ethical considerations

The study protocol was approved by the Human Investigations Committee of the Hospital of Chung Shan Medical University before the study started. Because all participating girls were 8 years old or younger, the parents or guardians of the participating girls uniformly provided signed informed consent.

### Bone age, body mass index (BMI)

Left-hand X-rays were performed on all subjects, and bone age (BA) was determined using the method of Greulich and Pyle [20]. Three replicate measurements of standing height were made using a wall-mounted stadiometer. BMI was calculated by dividing body weight (kg) by the square of height in meters (m^2^). Tanner stage standards were used to assess breast and pubic hair development [21, 22].

### Blood and data collection

After girls fasted eight-hours, blood specimens were collected for measurement of basal E2 (pmol/L), follicle stimulating hormone (FSH, U/L), LH (U/L), growth hormone (GH), IGF-1, and insulin-like growth factor binding protein 3 (IGFBP-3).

### GnRH test

After the subjects had fasted overnight, venous access was secured with a three-way stopcock and heparinized saline for the GnRH test. After baseline blood samples (2 mL) were drawn for LH & FSH or estradiol measurements, GnRH (range for children: 2.5 mcg/kg to 100 mcg/kg) was administered intravenously as a bolus. After 20 min and 60 min, blood samples (2 mL) for LH & FSH assessments were drawn and serum was harvested [23, 24].

### Immunoassays

Serum E2 levels were measured using a commercial radioimmunoassay kit (Diagnostic Systems Laboratories). Sensitivity was 2.2 pg/ml, with intra- and inter-assay coefficients of variation (CV) at 7.5 and 9.3%, respectively. Serum FSH and LH levels were measured by enzyme immunoassay (FSH: Abbott Laboratories, Rome, Italy; LH: Dade Behring, Milan, Italy). Sensitivity for both assays was 0.2 mIU/ml. Intra- and inter-assay CVs were 4.7 and 8.9%, respectively, for FSH, and 3.1 and 4.0%, respectively, for LH.

### Quantification of serum GH, IGF-1 and IGFBP-3 levels

After acid extraction, serum GH, IGF-I and IGFBP-3 levels were measured using the commercial radioimmunoassay (RIA) kits (Diagnostic Systems Laboratories, Webster, TX, USA). The sensitivities were 0.01 ng/mL, 0.9 mg/L and 0.01 ng/mL for GH, IGF-1 and IGFBP-3, respectively. The intra- and interassay CVs were 5.3 and 5.7% for GH; 7.2 and 9.8% for IGF-1; and 5.8 and 8.2% for IGFBP-3.

### Genomic DNA extraction

Venous blood from each subject was drawn into Vacutainer tubes containing EDTA and stored at 4 °C. Genomic DNA was extracted by QIAamp DNA Blood Mini Kits (Qiagen, Valencia, CA, USA) according to the manufacturer’s instructions. DNA was dissolved in Tris-EDTA buffer (10 mM Tris (pH 7.8) and 1 mM EDTA) and then quantitated by a measurement of optical density at 260 nm. The final preparation was stored at − 20 °C and used as a template for PCR.

### Polymerase chain reaction-restriction fragment length polymorphism (PCR-RFLP)

The IGF-1R, IGF-2, IGF-2R and INS gene polymorphisms were determined by PCR-RFLP assay. The sequences of primers used to amplify the related genotype and restriction enzyme for digestion as well as PCR products after digestion were listed in Table 1. PCR was performed in a 10 μL volume containing 100 ng DNA template, 1.0 μL 10´ PCR buffer (Invitrogen, Carslbad, CA, USA), 0.25 U Taq DNA polymerase (Invitrogen), 0.2 mM dNTPs (Promega, Madison, WI, USA), and 200 nM primer (MDBio Inc., Taipei, Taiwan). The PCR products of gene polymorphisms were subjected to enzymatic digestion by incubation with related restriction enzyme for 4 h at 37 °C and were then submitted to electrophoresis in 3% agarose gels.

**Table 1:** Sequences of primers used to amplify related genotypes, restriction enzymes for digestion, and sizes of PCR products after digestion

Gene name	Primers	Annealing temperature	Enzyme	Polymorphism
*IGF-1R* ^a^	5’-TGCTTTAATTACGGTTTCTTC-3’	60 °C	*Mnl* I	G:132, 77, 50, 20 bp
	5’-GCTTTTCAGGAACTTTCTCTT-3’			
				A:132, 97, 50 bp
*IGF-2*				
+ 3123^a^	5’-CCCCAGGTCACCCCATGTGA-3’	65 °C	*Apa*I	G: 173, 63 bp
	5’-GGGCTGGAGGCAGCTGAGTG −3’			A: 236 bp
+ 3580^a^	5’-CCACCCCTTCTGGGAAGCTAAAAG-3’	56 °C	*Msp*I	A: 122, 118 bp
	5’-CCCTCGGTCCTCCAGGAATGGACA-3’			G: 122, 118, 34 bp
*IGF-2R* ^a^	5’-AACAATGGTTAAAGCCGGATTG-3’	67 °C	*Nci I*	A: 456 bp
	5’-GGCCCGGGTGCAGCCAGGCACTG-3’			G: 307, 149 bp
*INS* ^a^	5’-GGGTCCCCTGCAGAAGCGTGGCA-3’	65 °C	*Pst*I	T: 562 bp
	5’-CTCCCTCCACAGGGACTCCATC-3’			C: 470, 92 bp
*IGF-1*				
+ 1770^b^	5′-tagaatattatttatagtattaaac [a/g]aggttttactagatatgtagtaact −3’	60 °C	−	T:VIC dye C:FAM dye
+ 6093^b^	5′-acagataaaagatgtaagtagacag [c/t]ttgaggtttcagagtccctcctgc − 3’	60 °C	−	G: VIC dye A: FAM dye
*IGFBP-3*				
-202^b^	5′-tcgcccgggcacctgctcctcgtgc [g/t]cacgcccggagcccgggtcaccttg	60 °C	−	C: VIC dye A: FAM dye

^a^using PCR-RFLP methods; ^b^using real-time PCR methods

### Real-time PCR

The *IGF-1 + 1770, + 6093,* and *IGFBP-3 -202* genes polymorphisms were determined by real-time PCR assay. Real-time PCR based on VIC (green) and FAM (blue) fluorescent dyes were applied for accurate quantification of the target sequence. The sequences of *IGF-1 + 1770, + 6093,* and *IGFBP-3 -202*-specific primers and PCR conditions were listed in Table 1. Strength of fluorescence for each sample was detected in each reaction cycle and plotted the fluorescent values against cycle number. The quantity and polymorphisms of each gene were observed by the intensity and color of fluorescence.

### Statistical analysis

Subjects’ demographics and characteristics data were represented as mean ± standard deviations (SD). The height, weight, and BMI values were used to calculate the z-scores with the WHO Child Growth Standards for subjects 5 years and younger [25] and the WHO 2007 reference for ages 5 years to 19 years [26]. The comparison of the subjects’ demographic and characteristic data between groups was performed using Mann-Whitney U test because the data were not normally distributed. Moreover, a Kruskall-Wallis test was conducted for to assess associations between two or more than two types of genotypes or combinations of genotypes and the subjects’ demographic and characteristics data. A Spearman correlation analysis was applied for identifying the correlation between serum IGF-1 and IGFBP-3 levels with subjects’ demographic and characteristic data; the coefficient of correlation r was calculated. A Pearson’s correlation was also applied for the correlation analysis with adjusted subjects’ BMI. All statistical analyses were carried out with IBM SPSS statistical software version 22 for Windows (IBM Corp., Armonk, NY, USA).

## Results

This study enrolled a total of 489 girls who were classified into the CPP (*n* = 264) and EP control (*n* = 225) groups. Demographics and characteristics of CPP and control groups are summarized in Table 2. The mean chronological age of all subjects was 8.61 yrs. (SD = 1.36) and was not significantly different between groups. There were significant differences between the CPP and control groups in most demographics and characteristics, except for GH levels (Table 2).

**Table 2:** Demographic and clinical characteristics of CPP and control groups

Variables	Total (*n* = 489)	Control group (*n* = 225)	CPP group (*n* = 264)	*p*-value
Chronological age (CA), years	8.61 ± 1.36	8.75 ± 1.55	8.49 ± 1.16	0.199
Bone age (BA), years	9.70 ± 1.98	8.75 ± 2.14	10.51 ± 1.39	< 0.001*
BA/CA ratio	1.13 ± 0.18	1 ± 0.17	1.24 ± 0.08	< 0.001*
Age of onset, years	7.63 ± 1.18	7.99 ± 1.44	7.32 ± 0.77	< 0.001*
Height, cm	136.20 ± 10.76	131.3 ± 10.07	140.38 ± 9.5	< 0.001*
Z-scores of height^a^	130.12 ± 8.24	0.09 ± 1.02	1.86 ± 1.40	0.078
Weight, kg	34.00 ± 9.44	30.44 ± 8.7	37.03 ± 8.99	< 0.001*
Z-scores of weight^a^	26.26 ± 3.24	26.26 ± 3.11	26.71 ± 3.33	0.137
BMI	18.02 ± 3.14	17.4 ± 3.3	18.55 ± 2.91	< 0.001*
Z-scores of BMI^a^	16.02 ± 0.58	16.10 ± 0.72	15.96 ± 0.41	0.008*
E2, pmol/l	32.74 ± 35.39	32.07 ± 48.24	33.31 ± 18.58	< 0.001*
FSH, U/l	14.40 ± 9.23	10.12 ± 8.03	18.05 ± 8.61	< 0.001*
LH, U/l	23.54 ± 30.70	5.62 ± 12.61	38.82 ± 33.23	< 0.001*
GH, ng/ml	3.43 ± 5.05	3.3 ± 5.22	3.55 ± 4.91	0.320
IGF-1, ng/ml	321.56 ± 125.87	257.46 ± 104.08	376.18 ± 116.88	< 0.001*
IGFBP-3, ng/ml	2282.02 ± 1167.12	1873.12 ± 1441.39	2630.51 ± 702.17	< 0.001*

*p < 0.05

^a^Z-scores calculated with WHO Child Growth Standards for children up to 5 years old [25] and WHO 2007 reference for children older than 5 years [26]

We assessed the correlation of serum IGF-1 (Table 3) and IGFBP-3 (Table 4) levels with subjects’ characteristics. The serum IGF-1 level in the total population was positively correlated with most characteristics, especially bone age and LH. The IGF-1 levels in the CPP group showed a higher correlation with bone age, z-scores of height, z-scores of weight, z-scores of BMI, and LH compared to the control group. After adjusting for subjects’ BMI which differed significantly between the control *a*nd CPP groups, the IGF-1 levels in the CPP group also showed a higher correlation with bone age, CPP onset age, z-scores of height and weight, and LH levels compared to the control group (Table 3). The correlations between IGF-1 levels and the different demographic and pathological features after adjusting for bone age are summarized in Table 5.

**Table 3:** Correlations of IGF-1 levels with demographic and pathological features with and without BMI adjustment by group

Variables	r with IGF1 (ng/ml)			^a^Adjusted r’ with IGF1 (ng/ml)		
	Total (*n* = 489)	Control (*n* = 225)	CPP (*n* = 264)	Total (*n* = 485)	Control (*n* = 221)	CPP (*n* = 261)
Chronological age, years	0.275^c^	0.276^c^	0.466^c^	0.247^c^	0.259^c^	0.434^c^
Bone age, years	0.429^c^	0.172^c^	0.423^c^	0.386^c^	0.159^e^	0.390^c^
BA/CA ratio	0.292^c^	−0.015	−0.113	0.253^c^	−0.023	− 0.113
Age of onset, years	−0.019	0.141^e^	0.224^c^	0.053	0.225^d^	0.266^c^
Z-scores of height^a^	0.276^c^	0.160^e^	0.486^c^	0.289^c^	0.147^e^	0.411^c^
Z-scores of weight^a^	0.293^c^	0.151	0.475^c^	0.299^c^	0.120	0.435^c^
Z-scores of BMI^a^	0.263^c^	0.157^e^	0.464^c^	–	–	–
E2, pmol/l	0.165^c^	0.165^e^	0.273^c^	0.159^c^	0.164^e^	0.254^c^
FSH, U/l	0.045	−0.283^c^	−0.136^e^	0.037	−0.303^c^	− 0.115
LH, U/l	0.523^c^	0.172^c^	0.447^c^	0.496^c^	0.101	0.425^c^
GH, ng/ml	0.176^d^	0.281^d^	0.111	0.199^c^	0.297^c^	0.131^e^

^a^Adjusted r’: correlation with adjustment for BMI

^b^Z-scores calculated with WHO Child Growth Standards for children up to 5 years old [25] and WHO 2007 reference for children older than 5 years [26]

^c, d, e^Correlations are significant at the 0.001, 0.01, and 0.05 levels (2-tailed)

**Table 4:** Correlations of IGFBP-3 levels with demographic and pathological features with and without BMI adjustment by group

Variables	r with IGFBP-3 (ng/ml)			Adjusted r’ with IGFBP-3 (ng/ml)^a^		
	Total (*n* = 489)	Control (*n* = 225)	CPP (*n* = 264)	Total (*n* = 485)	Control (*n* = 221)	CPP (*n* = 261)
Chronological age, years	0.096^e^	0.148^e^	0.110	0.077	0.134^e^	0.100
Bone age, years	0.343^c^	0.266^c^	0.157^e^	0.314^c^	0.240^c^	0.148^e^
BA/CA ratio	0.360^c^	0.227^d^	0.096	0.335^c^	0.199^d^	0.098
Age of onset, years	−0.057	0.170^e^	−0.033	−0.102^e^	−0.053	0.009
Z-scores of height^b^	0.131^e^	0.171^e^	0.121^e^	0.012	−0.049	0.087
Z-scores of weight^b^	0.078	0.019	0.103	0.028	−0.023	0.087
Z-scores of BMI^b^	0.124^d^	0.170^e^	0.102	–	–	–
E2, pmol/l	0.042	0.043	0.018	0.036	0.041	0.012
FSH, U/l	0.395^c^	0.584^c^	−0.106	0.395^c^	0.593^c^	−0.102
LH, U/l	0.262^c^	0.274^c^	0.066	0.240^c^	0.308^c^	0.057
GH, ng/ml	0.026	−0.018	0.090	0.036	−0.005	0.093

^a^Adjusted r’: correlation with BMI adjustment

^b^Z-score calculated with WHO Child Growth Standards for children up to 5 years old [25] and WHO 2007 reference for children older than 5 years [26]

^c, d, e^Correlations are significant at the 0.001, 0.01, and 0.05 levels (2-tailed)

**Table 5:** Correlations between IGF-1 levels and demographic and pathological features after adjusting for bone age by group

Variables	^a^r” with IGF1 (ng/ml)		
	Total (*n* = 489)	Control (*n* = 225)	CPP (*n* = 264)
Chronological age, years	0.016	0.143^e^	0.204^d^
Bone age, years	–	–	–
BA/CA ratio	−0.035	− 0.118	− 0.195^d^
Age of onset, years	− 0.170	0.143^e^	− 0.067
Z-scores of height^b^	0.021	0.145^e^	0.185^d^
Z-scores of weight^b^	0.041	0.109	0.244^c^
Z-scores of BMI^b^	0.069	0.015	0.233^c^
E2, pmol/l	0.112^e^	−0.141^e^	0.160^e^
FSH, U/l	0.049	−0.297^c^	0.040
LH, U/l	0.419^c^	−0.222^c^	0.362^c^
GH, ng/ml	0.159^d^	0.221^d^	0.080

^a^Adjusted r”: correlation with adjustment for BA (bone age)

^b^Z-scores calculated with WHO Child Growth Standards for children up to 5 years old [25] and WHO standards for children older than 5 years [26]

^c,d,e^Correlations are significant at the 0.001, 0.01, and 0.05 levels (2-tailed)

The serum IGFBP-3 level of the total population was significantly and positively correlated to bone age, BA/CA ratio, and FSH (Table 4). The serum IGFBP-3 level was positively correlated to FSH in the control group but not in the CPP group (Table 4), both prior to and after adjusting for the subjects’ BMI (Table 4).

There were no significant differences in the distribution of genotypes and allele frequency of *IGF1R, IGF-1(6093), IGF-1(1770), IGF-2(3123), IGF2R, IGF-2(3580), IGFBP-3*, and insulin between the control and CPP groups (Additional file 1: Table S1). There were also no significant differences in genotype distribution of combinations of two SNPs in two distinct genes between the CPP and control groups (Additional file 1: Tables S2–1; S2–2).

**Fig. 1:**
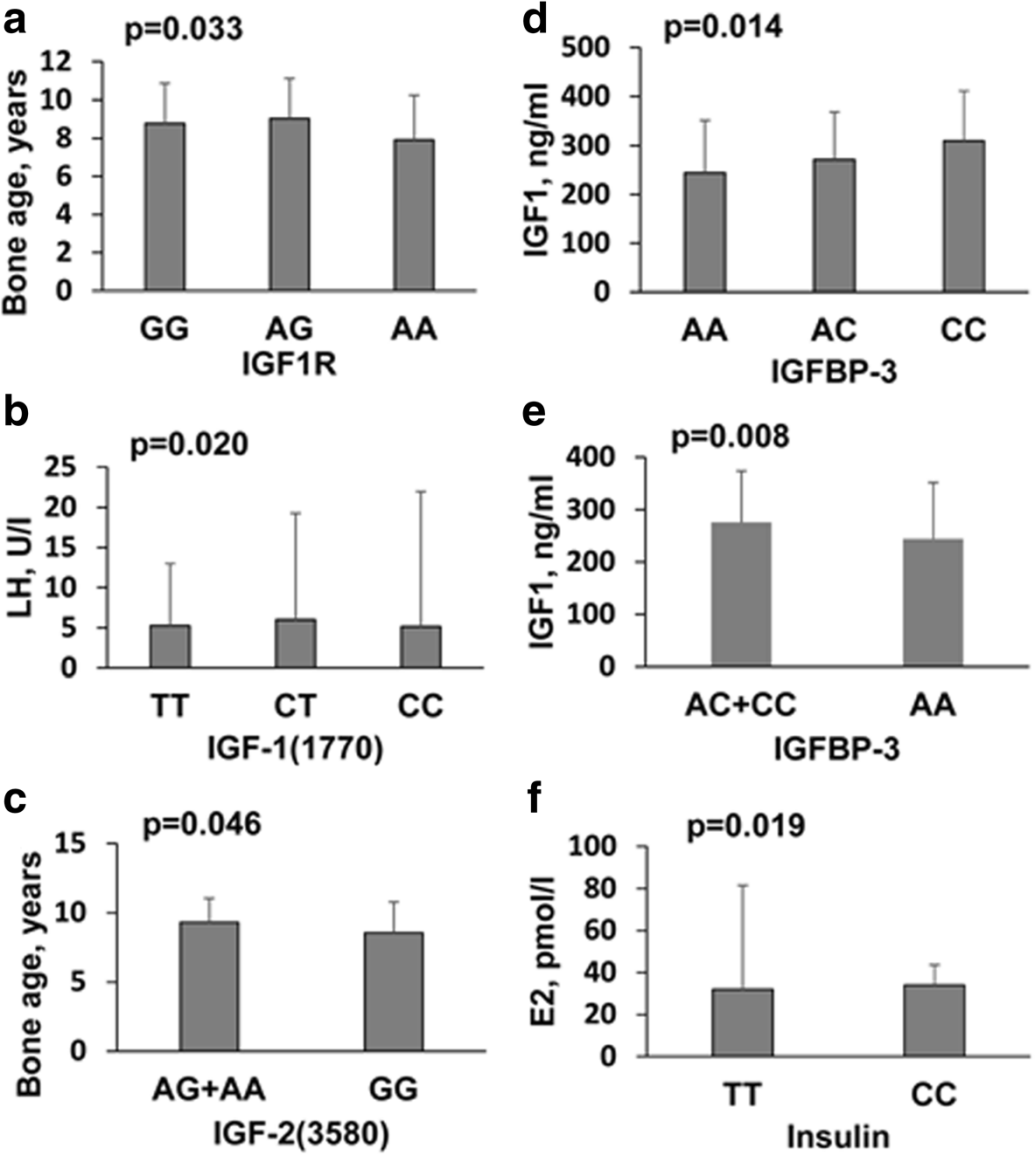
Data were presented as mean +-SD given SNP genotype (**a**. Bone age v.s IGF1R; **b**. LH v.s IGF-1(1770); **c**. Bone age v.s IGF-2(3580); **d**. IGF-1 v.s IGFBP-3(AA, AC, CC); **e**. IGF-1 v.s IGFBP-3(AC+CC, AA); **f**. E2 v.s Insulin)

**Fig. 2:**
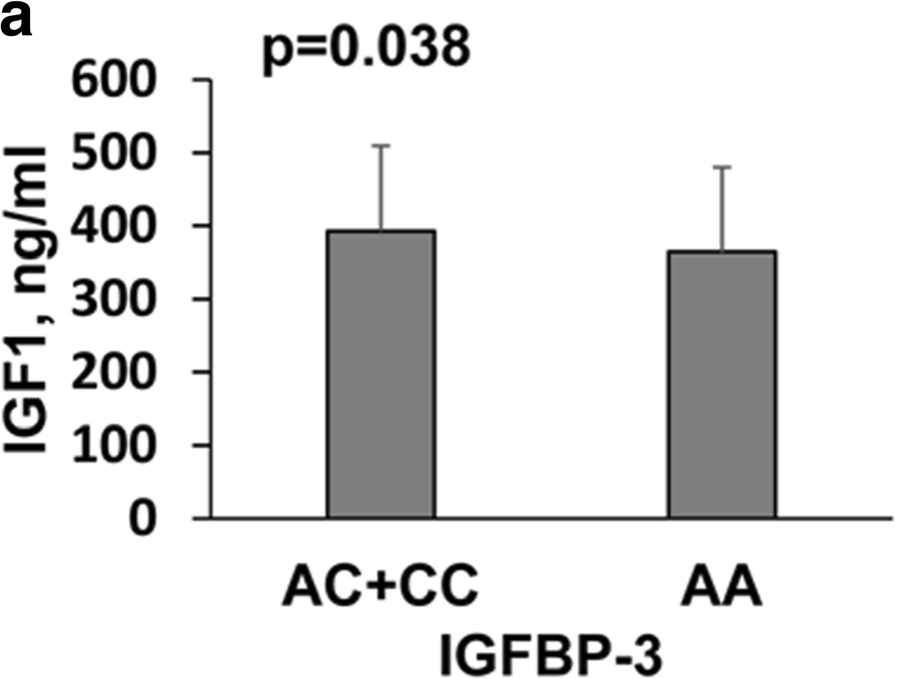
Demographic and pathological features significantly associated with SNP genotype in CPP group. Data were presented as mean ± SD given SNP genotype and p-value was presented form difference among genotypes

We evaluated the association of demographic and pathological features with SNP genotype in both the control and CPP groups. In the control group, bone age was significantly associated with *IGF1R* (*p* = 0.033) and *IGF-2(3580)* (*p* = 0.046); E2 levels were significantly associated with insulin (*p* = 0.019); and the LH levels were significantly associated with *IGF-1(1770)* (*p* = 0.020). IGF-1 levels were significantly associated with IGFBP-3 (*p* = 0,014) (Fig. 1; Additional file 1: Table S3–1). IGF-1 levels were significantly associated with IGFBP-3 in the CPP group (*p* = 0.038) (Fig. 2; Additional file 1: Table S3–2).

**Fig. 3:**
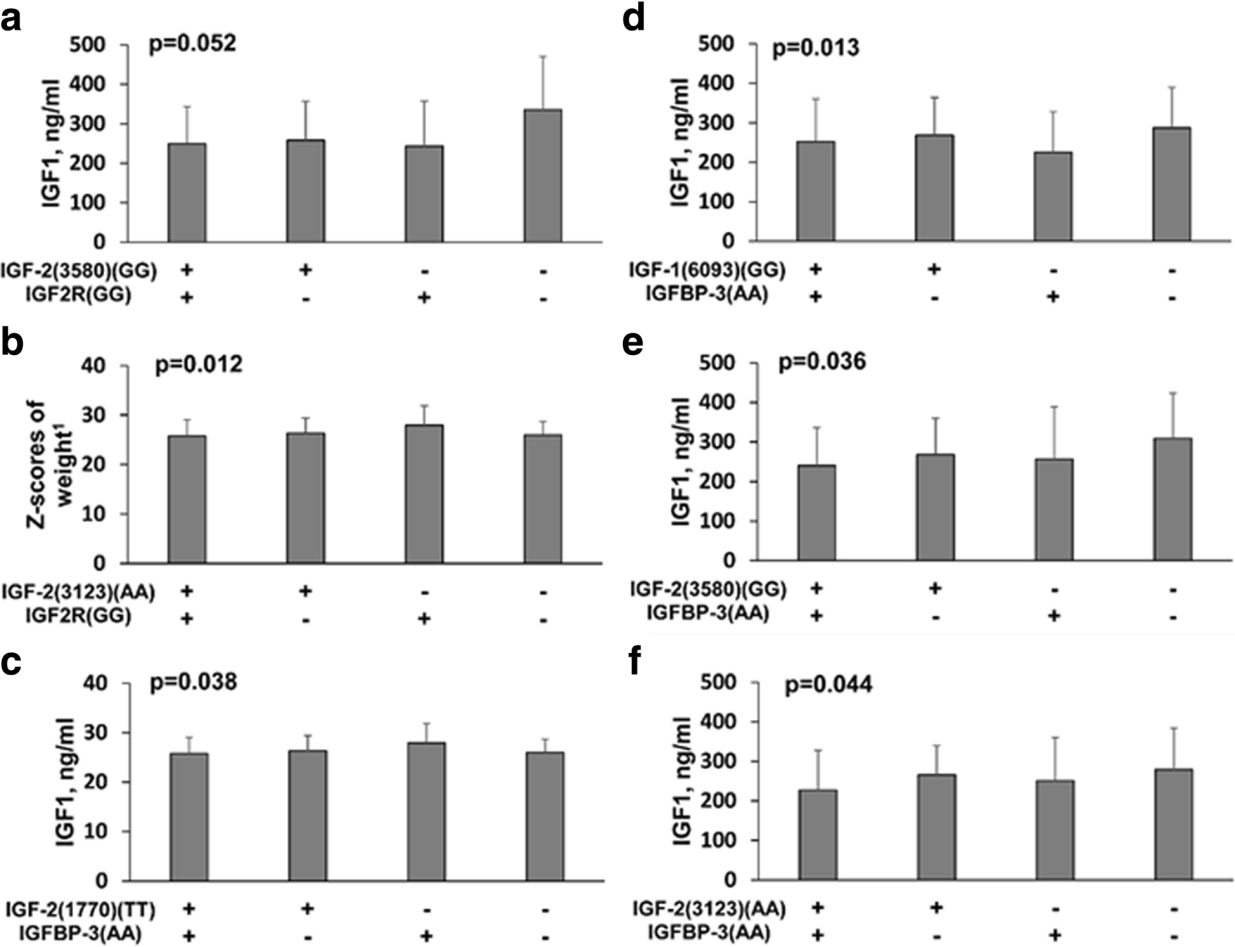
Demographic and pathological features significantly associated with a combination of two SNP genotypes in the control group. Data were presented as mean ± SD for a given two combination of SNP genotypes. (**a**. IGF-1 v.s IGF-2(3580)(GG), IGF-2R(GG); **b**. Z score of weight v.s IGF-2 (3123)(AA), IGF2R(GG); **c**. IGF-1 v.s IGF-2 (1770)(TT), IGFBP-3(AA); **d**. IGF-1 v.s IGF-1(6093)(GG), IGFBP-3(AA); **e**. IGF-1 v.s IGF-2(3580)(GG), IGFBP-3(AA); **f**. IGF-1 v.s IGF-1(3123(AA), IGFBP-3(AA))

**Fig. 4:**
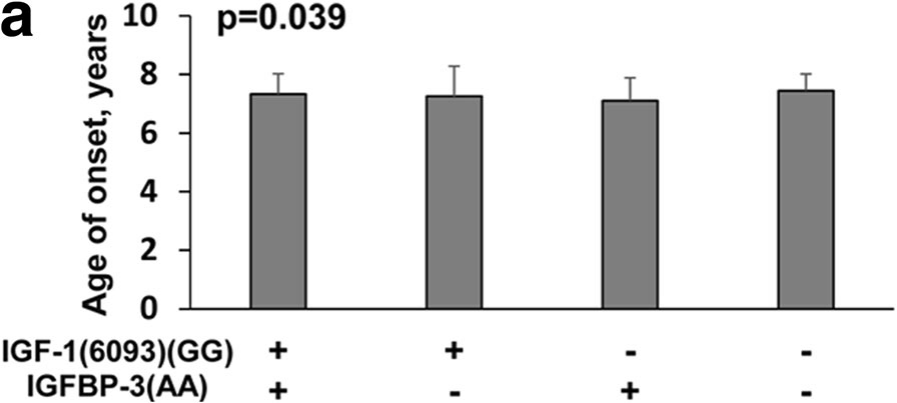
Demographic and pathological features significantly associated with a combination of two SNP genotypes in the CPP group. Data were presented as mean ± SD for a given combination of two SNP genotypes. *p*-value was presented for differences among genotypes

We compared the association of demographic and pathological features with a combination of two genotypes in the control (Fig. 3; Additional file 1: Table S4–1) and CPP (Fig. 4; Additional file 1: Table S4–2) groups. For the control group, the serum IGF-1 level was associated with the combinations of [*IGF-1(1770) + IGFBP-3*] (p = 0.038), [*IGF-1(6093) + IGFBP-3*] (*p* = 0.013), and [*IGF-2(3580) + IGFBP-3*] (*p* = 0.036). The z-scores of BMI were associated with the combination, [*IGF*-*2(3123)* + IGF*-2R*] (*p* = 0.012) (Fig. 3). For CPP group, the age of onset was shown associated with combination [*IGF-2(6093) + IGFBP-3*] (*p* = 0.039) (Fig. 4). However, none of the other demographic or pathological features were significantly associated with the combination of two SNP genotypes (Additional file 1: Table S4–2).

**Fig. 5:**
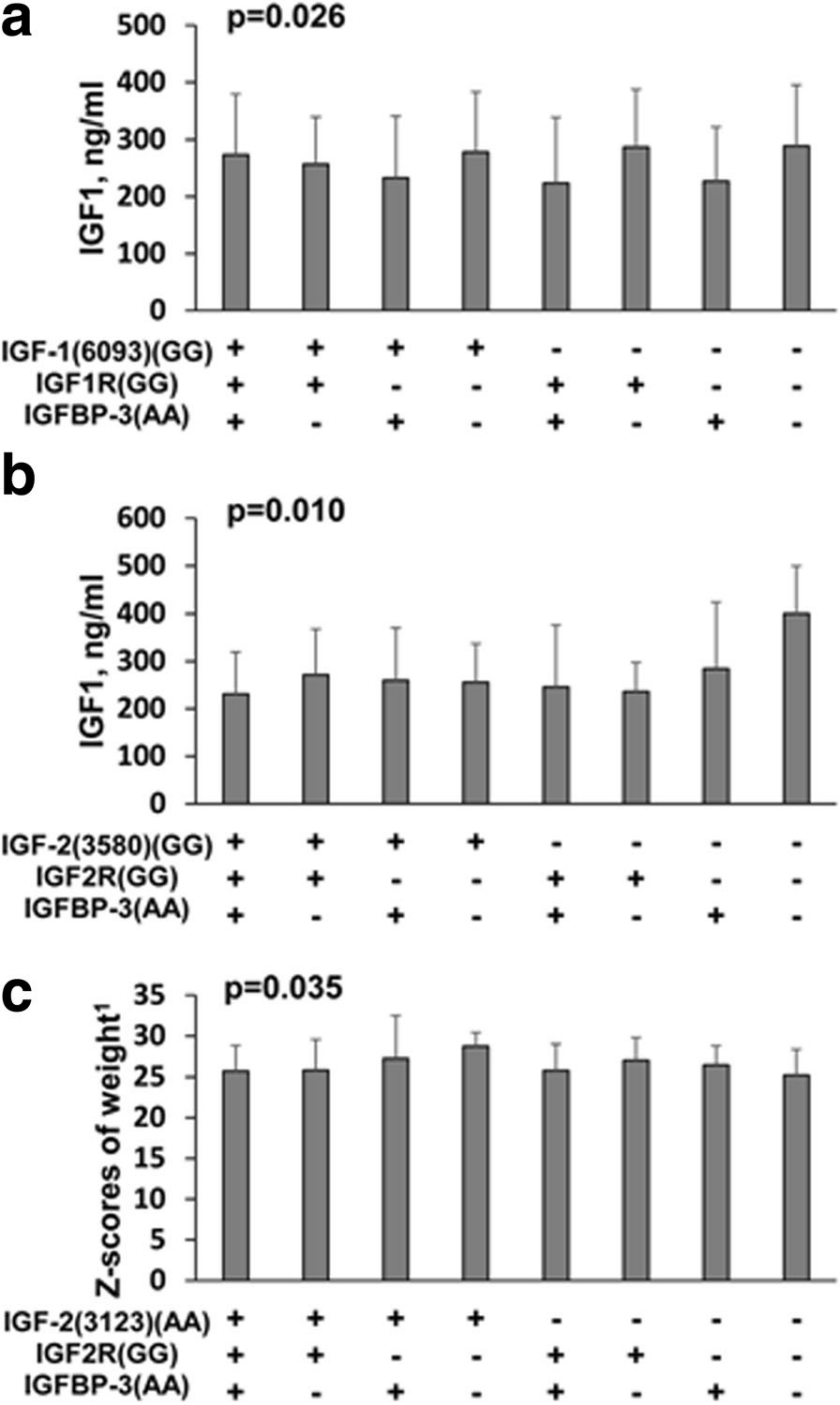
Demographic and pathological features significantly associated with a combination of three SNP genotypes in the control group. Data were presented as mean ± SD for the given combination of three SNP genotypes. (**a**. IGF-1 v.s IGF-1(6093)(GG), IGF-1R(GG), IGFBP-3(AA); **b**. IGF-1 v.s IGF-2(3580)(GG), IGF-2R(GG), IGFBP-3(AA); **c**. Z score of weight v.s IGF-2(3123)(AA), IGF-2R(GG), IGFBP-3(AA))

**Fig. 6:**
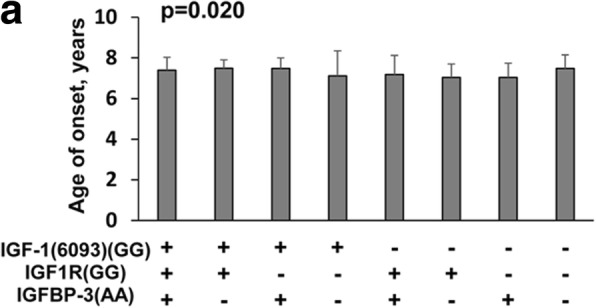
Demographic and pathological features significantly associated with a combination of three SNP genotypes in the CPP group. Data were presented as mean ± SD for a given combination of three SNP genotypes. *p*-value was presented for difference among genotypes

Furthermore, we compared the association between demographic and pathological features and a combination of the *IGFBP-3* genotypes with two additional genes, in the control (Additional file 1: Table S5–1) and CPP (Additional file 1: Table S5–2) groups. In the control group, there was a significant association between IGF-1 levels and the combination, [*IGF-1(6093) + IGF1R + IGFBP-3*] (*p* = 0.026), and [*IGF-2(3580) + IGF2R + IGFBP-3*] (*p* = 0.010). The z-scores of weight was associated with the gene combination, [*IGF-2(3123) + IGF2R + IGFBP-3*] (*p* = 0.035) (Additional file 1: Table S5–1; Fig. 5). The CPP group showed a significant association between age of onset and the combination [*IGF-2(3580) + IGF2R + IGFBP-3*] (*p* = 0.020; Fig. 6). However, none of the other demographic or pathological features were significantly associated with the combination of three SNP genotypes (Additional file 1: Table S5–2).

## Discussion

Our findings showed that the IGF-1 serum levels of the CPP group exhibited a higher correlation with bone age, z-scores of height, z-scores of weight, and LH than those of the control group, regardless of adjustment for BMI. The height of the CPP group, but not the z-score of the height, was associated with *IGF-2(3580).* The BMI of the CPP group, but not the z-score of the BMI, was associated with *IGF-2(3580)*, *IGF1R*, and the combinations of [*IGF-2(3580) + IGF2R*], and [*IGF-2(3580) + IGFBP-3*]. The weight of the CPP group, but not the z-score of the weight, was associated with the combination of [*IGF-2(3580) + IGFBP-3*] (*p* = 0.024). The CPP group showed a significant association between weight and BMI with the combination of [*IGF-2(3580) + IGF2R + IGFBP-3*]. The distribution of genotypes and allele frequency in *IGF1R, IGF-1(6093), IGF-1(1770)*, *IGF-2(3123), IGF2R, IGF-2(3580), IGFBP-3*, and *insulin* between control and CPP groups were similar. Likewise, the distributions of the gene SNP combinations were similar.

A number of studies have previously investigated the relationship between various IGF family SNPs and variables such as age, BMI, and weight. The IGF family plays an important role in stimulating skeletal growth, cell differentiation and metabolism, and has been shown to influence body composition [27]. IGF-2 has been reported to play a role in fetal development, while IGF-1 is expressed after birth [28]. A polymorphism in the IGF-1 promoter was reported to be associated with IGF-1 serum levels, birth weight and body height in girls with CPP, as well as in adults. This polymorphism was also shown to be associated with higher body weight, BMI, fat mass, and waist circumference in young subjects [27]. A SNP in IGF-1R was shown to influence free IGF-1 plasma concentrations. This A → G variant was predicted to generate a silent mutation, E1013E, and was associated with higher IGF-1 concentrations in Italian adults [29]. Homozygosity for the A variant was associated with the lowest mean IGF-1 concentration, whereas heterozygosity was associated with a slightly higher mean IGF-1 concentration. Homozygosity for the G variant was associated with the highest mean IGF-1 concentration. These data suggested that IGF-1 concentrations were influenced by the IGF-1R genotype at codon 1013 [29].

The AA, AG, and GG have been reported there have a dramatic difference in birth weight standard deviation scores (SDSs) in neonatal + 3123/ApaI genotypes of IGF-2 [30]. AA homozygotes had a mean birth weight SDS of 0.18 lower than that of GG homozygotes (*p* = 0.01), while heterozygotes showed an intermediate mean value. In contrast, there was no significant difference in birth weight SDSs between the AA, AG and GG maternal + 3123/ApaI genotypes. There was also no significant difference in birth weight SDSs between the AA, AG, and GG + 3580/MspI genotypes in both neonatal and maternal samples. However, the association between IGF-2 polymorphisms and size at birth remains controversial [30]. Analysis of the + 3123/ApaI genotype in 693 Hertfordshire adults showed that birth weight was highest for the GG genotype but the differences were not statistically significant [31]. Other data showed that the IGF-2 genotype was not significantly associated with BMI and/or birth weight in 294 healthy volunteers, but there was a statistically significant correlation between birth weight and BMI in subjects with the GG genotype whose birth weight was higher than 3.5 kg [32]. One reason for these different findings regarding the association between + 3123/ApaI polymorphism and size at birth may be differences in race and size of the study population, or differences in handling of the somatoscopic characteristics [32].

A recent study found a significant difference between the experimental and control groups in the distribution frequency of the IGF-2 + 3580 polymorphism. Additionally, multiple regression model analyses showed that the presence of the IGF-2R AA or AG genotypes may exert a protective effect against hepatitis C [odds ratio (OR) = 0.35, 95% confidence interval (CI) = 0.15–0.82]. The combination of IGF-2 + 3580 AA genotype and IGF-2R GG genotype may be associated with a significantly lower risk of HCC (OR = 0.20, 95% CI = 0.05–0.87). No polymorphisms of any IGF genes were associated with liver-related clinicopathological markers in serum [33]. Comparison of allele frequencies between the premature pubarche (PP), hyperandrogenism (HA), and healthy control subjects showed a significantly higher frequency of the G allele in the PP group compared to the other groups, (*P* = .0781). However, allele frequencies were comparable in the HA and the healthy control subjects [34].

IGF-1 levels are significantly higher in girls of any age undergoing puberty than those in prepubertal girls. Our results suggest that the known variants in the genes of the IGF-1 axis play minor roles in the timing of elevation of IGF-1 and IGFBP-3 protein levels during puberty, similar to the role that SNPs in IGF-1 axis genes play in breast cancer [35]. In a similar manner, although the CPP group had significantly higher leptin levels than the control group, the differences in timing and expression level of leptin could not be explained by single nucleotide polymorphisms in either leptin or the leptin receptor [36].

The similar distribution of SNPs of the genes in the IGF-1 axis between CPP and control groups were in contrast to the skewed distribution of mutations and SNPs of four genes that were more prevalent in patients with CPP: the autosomal dominant *GPR54 R386P* mutation [37, 38], several polymorphisms (55,648,184; 55,648,186) in the *KISS1* gene [39], the intron 4 (TTTA)_13_ repeat in the cytochrome *P450 19A1* gene *CYP19A1* gene [40], and a haplotype in the 5′ promoter region of the *LH* β gene [41]. In addition, eight unrelated girls with CPP at age 6 had one of five novel heterozygous loss-of-function mutations in the makeorin ring finger 3 (*MKRN3*), which normally suppresses or delays GnRH secretion [42]. Conversely, the mutations and SNPs of four genes were less prevalent in patients with CPP than in controls: the polymorphism (55648176) in the *KISS1* gene [39], the AC haplotype of *Lin28B* in two positions (SNPs *rs4946651, RS369065*) [43], and the cytochrome P450 *CYP1B1 Eco571* variant (V432 L) [44]. In addition, leptin levels were significantly higher in the CPP group than those in the control group but SNPs in either the leptin receptor or leptin genes were not able to explain the differences [36]. Taken together, these studies indicate that multiple genes can influence the onset of puberty, and specific genotypes and haplotypes can increase the risk for development of CPP [39–43].

In our present study, we found no significant association between the SNPs evaluated and z-scores of height, weight, or BMI in either the EP or CPP groups. However, our data showed that the bone ages of subjects in the IGF-1R + 1013 (AG) and IGF-2 + 3580 (AG + AA) groups were more advanced in the EP group. This could possibly be because although the girls did not appear to have entered puberty, their bone age had already acquired the characteristics of puberty. It is possible that differences between our data and previous studies could be due to ethnic or other demographic differences in our study population. Although our data did not directly prove that IGF-1R and IGF-2 + 3580 were related to precocious puberty in girls, our results showed that the IGF-1R G variant and the IGF-2 + 3580 A variant were associated with CPP. In addition, we also believe that the interaction between IGF-I and IGF-II polymorphisms could play an important role, and warrants further investigation.

Some important limitations of this study were 1) healthy subjects were not included, 2) IGF-2 levels were not measured, and 3) the role of other genetic pathways which could play a role in CPP were not investigated.

## Conclusion

In conclusion, specific genotypes from several genes (GPR54, *KISS, CYP19A1, and Lin28B)* can accelerate or slow the onset of puberty and have been associated with higher or lower prevalence in girls with CPP. The IGF-1 protein levels coupled with human GH levels affect timing of menarches [45–47]. This study showed that single SNPs of the genes in the IGF-1 axis (*IGF-1(6093), IGF-1(1770), IGF1R, IGF-2(3123), IGF-2(3580), IGF2R, IGFBP-3(− 202)*) did not appear to exert a significant role in the risk for development of CPP. However, several combinations were significantly associated with higher IGF-1 blood levels. Whether epigenetic modulation of the genes in the IGF axis plays a more prominent role in the risk for CPP than SNPs will require further research. Alternatively, other genes [37–40] or environmental factors [48, 49]) appear to play a more prominent role in triggering the development of CPP.

## Additional file

Additional file 1: **Table S1.** Distribution of SNPs in *IGF1R, IGF-1(6093), IGF-1(1770), IGF-2(3123), IGF2R, IGF-2(3580), IGFBP-3*, and *insulin* in CPP and control groups. **Table S2–1** Summary of genotype distribution of two SNP combinations in two distinct genes by group. **Table S2–2.** (continued) Summary of genotype distribution in two SNP combinations in two distinct genes by group. **Table S3–1.** Associations between demographic and pathological features and SNP genotypes in control group. **Table S3–2.** Associations between demographic and pathological features and SNP genotypes in CPP group. **Table S4–1.** Comparison of associations between demographic and pathological features with two SNP genotype combinations in the control group. **Table S4–2.** Associations between demographic and pathological features and two SNP genotype combinations in the CPP group. **Table S5–1.** Associations between demographic and pathological features and combinations of IGFBP-3 and two additional genes in the control group. **Table S5–2.** Associations between demographic and pathological features and combinations of IGFBP-3 and two additional genes in the CPP group. **Table S6.** Summary of the power for given control and CPP groups. (DOCX 144 kb)

## Abbreviations

BA: Bone age
BMI: Body mass index
CPP: Central precocious puberty
CV: Coefficients of variation
E2: Estradiol
EP: Early puberty
FSH: Follicle stimulating hormone
GH: Growth hormone
GnRH: Gonadotropin-releasing hormone
IGF-1: Insulin-like growth factor-1
IGF-1R: Insulin-like growth factor 1 receptor
IGFALS: Insulin-like growth factor binding protein, acid labile subunit
IGFPB3: Insulin-like growth factor-binding protein 3
IRs: Insulin receptors
LH: Luteinizing hormone
LHRH: Luteinizing hormone-releasing hormone
MKRN3: Makeorin ring finger 3
PCR: Polymerase chain reaction
PP: Precocious puberty
PPP: Pseudo or peripheral precocious puberty
RFLP: Restriction fragment length polymorphism
RIA: Radioimmunoassay
SD: Standard deviation
