# Supplementary material for: Associations among IGF-1, IGF2, IGF-1R, IGF-2R, IGFBP-3, insulin genetic polymorphisms and central precocious puberty in girls
Source: BMC Endocr Disord. 2018 Sep 19;18:66. doi: 10.1186/s12902-018-0271-1 (PMC6154940; doi:10.1186/s12902-018-0271-1)
Supplement: Supplementary file 1 — Table S1. Distribution of SNPs in IGF1R, IGF-1(6093), IGF-1(1770), IGF-2(3123), IGF2R, IGF-2(3580), IGFBP-3, and insulin in CPP and control groups. Table S2–1 Summary of genotype distribution of two SNP combinations in two distinct genes by group. Table S2–2. (continued) Summary of genotype distribution in two SNP combinations in two distinct genes by group. Table S3–1. Associations between demographic and pathological features and SNP genotypes in control group. Table S3–2. Associations between demographic and pathological features and SNP genotypes in CPP group. Table S4–1. Comparison of associations between demographic and pathological features with two SNP genotype combinations in the control group. Table S4–2. Associations between demographic and pathological features and two SNP genotype combinations in the CPP group. Table S5–1. Associations between demographic and pathological features and combinations of IGFBP-3 and two additional genes in the control group. Table S5–2. Associations between demographic and pathological features and combinations of IGFBP-3 and two additional genes in the CPP group. Table S6. Summary of the power for given control and CPP groups. (DOCX 144 kb) [file 12902_2018_271_MOESM1_ESM.docx]

**Additional file 1**

**Table S1** Distribution of SNPs in *IGF1R, IGF-1(6093), IGF-1(1770), IGF-2(3123), IGF2R, IGF-2(3580), IGFBP-3*, and *insulin* in CPP and control groups.

| **Gene** | **Type** | **Total^1^**  **(n=489)** | **Control^1^**  **(n=225)** | **CPP^1^**  **(n=264)** | **p-value** |
| --- | --- | --- | --- | --- | --- |
| *IGF1R* | GG | 214 (43.8) | 108 (48) | 106 (40.2) | 0.056 |
|  | AG | 211 (43.1) | 84 (37.3) | 127 (48.1) |  |
|  | AA | 64 (13.1) | 33 (14.7) | 31 (11.7) |  |
|  |  |  |  |  |  |
|  | AG+AA | 275 (56.2) | 117 (52) | 158 (59.8) | 0.081 |
|  | GG | 214 (43.8) | 108 (48) | 106 (40.2) |  |
|  |  |  |  |  |  |
|  | A | 339 (34.7) | 150 (33.3) | 189 (35.8) | 0.420 |
|  | G | 639 (65.3) | 300 (66.7) | 339 (64.2) |  |
|  |  |  |  |  |  |
| *IGF-1(6093)* | GG | 340 (69.5) | 152 (67.6) | 188 (71.2) | 0.662 |
|  | AG | 138 (28.2) | 68 (30.2) | 70 (26.5) |  |
|  | AA | 11 (2.2) | 5 (2.2) | 6 (2.3) |  |
|  |  |  |  |  |  |
|  | AG+AA | 149 (30.5) | 73 (32.4) | 76 (28.8) | 0.381 |
|  | GG | 340 (69.5) | 152 (67.6) | 188 (71.2) |  |
|  |  |  |  |  |  |
|  | A | 160 (16.4) | 78 (17.3) | 82 (15.5) | 0.447 |
|  | G | 818 (83.6) | 372 (82.7) | 446 (84.5) |  |
|  |  |  |  |  |  |
| *IGF-1(1770)* | TT | 145 (29.7) | 68 (30.2) | 77 (29.2) | 0.696 |
|  | CT | 249 (50.9) | 117 (52.0) | 132 (50) |  |
|  | CC | 95 (19.4) | 40 (17.8) | 55 (20.8) |  |
|  |  |  |  |  |  |
|  | CT+CC | 344 (70.3) | 157 (69.8) | 187 (70.8) | 0.799 |
|  | TT | 145 (29.7) | 68 (30.2) | 77 (29.2) |  |
|  |  |  |  |  |  |
|  | C | 439 (44.9) | 197 (43.8) | 242 (45.8) | 0.519 |
|  | T | 539 (55.1) | 253 (56.2) | 286 (54.2) |  |
|  |  |  |  |  |  |
| *IGF2R* | GG | 342 (69.9) | 158 (70.2) | 184 (69.7) | 0.886 |
|  | AG | 125 (25.6) | 58 (25.8) | 67 (25.4) |  |
|  | AA | 22 (4.5) | 9 (4.0) | 13 (4.9) |  |
|  |  |  |  |  |  |
|  | AG+AA | 147 (30.1) | 67 (29.8) | 80 (30.3) | 0.900 |
|  | GG | 342 (69.9) | 158 (70.2) | 184 (69.7) |  |
|  |  |  |  |  |  |
|  | A | 169 (17.3) | 76 (16.9) | 93 (17.6) | 0.765 |
|  | G | 809 (82.7) | 374 (83.1) | 435 (82.4) |  |
|  |  |  |  |  |  |
| *IGF-2(3123)* | AA | 137 (28.0) | 60 (26.7) | 77 (29.2) | 0.795 |
|  | AG | 247 (50.5) | 117 (52.0) | 130 (49.2) |  |
|  | GG | 105 (21.5) | 48 (21.3) | 57 (21.6) |  |
|  |  |  |  |  |  |
|  | AG+GG | 352 (72) | 165 (73.3) | 187 (70.8) | 0.540 |
|  | AA | 137 (28) | 60 (26.7) | 77 (29.2) |  |
|  |  |  |  |  |  |
|  | A | 521 (53.3) | 237 (52.7) | 284 (53.8) |  |
|  | G | 457 (46.7) | 213 (47.3) | 244 (46.2) |  |
|  |  |  |  |  |  |
| *IGF-2(3580)* | GG | 389 (79.6) | 171 (76) | 218 (82.6) | 0.180 |
|  | AG | 97 (19.8) | 52 (23.1) | 45 (17.0) |  |
|  | AA | 3 (0.6) | 2 (0.9) | 1 (0.4) |  |
|  |  |  |  |  |  |
|  | AG+AA | 100 (20.4) | 54 (24.0) | 46 (17.4) | 0.072 |
|  | GG | 389 (79.6) | 171 (76.0) | 218 (82.6) |  |
|  |  |  |  |  |  |
|  | A | 103 (10.5) | 56 (12.4) | 47 (8.9) | 0.072 |
|  | G | 875 (89.5) | 394 (87.6) | 481 (91.1) |  |
|  |  |  |  |  |  |
| *IGFBP-3* | AA | 288 (58.9) | 132 (58.7) | 156 (59.1) | 0.794 |
|  | AC | 176 (36) | 83 (36.9) | 93 (35.2) |  |
|  | CC | 25 (5.1) | 10 (4.4) | 15 (5.7) |  |
|  |  |  |  |  |  |
|  | AC+CC | 201 (41.1) | 93 (41.3) | 108 (40.9) | 0.924 |
|  | AA | 288 (58.9) | 132 (58.7) | 156 (59.1) |  |
|  |  |  |  |  |  |
|  | A | 752 (76.9) | 347 (77.1) | 405 (76.47) | 0.881 |
|  | C | 226 (23.1) | 103 (22.9) | 123 (23.3) |  |
|  |  |  |  |  |  |
| *Insulin* | TT | 460 (94.1) | 210 (93.3) | 250 (94.7) | 0.525 |
|  | CT | 29 (5.9) | 15 (6.7) | 14 (5.3) |  |
|  |  |  |  |  |  |
|  |  |  |  |  |  |
|  | C | 29 (3.0) | 15 (3.3) | 14 (2.7) | 0.531 |
|  | T | 949 (97.0) | 435 (96.7) | 514 (97.3) |  |

^1^Data are represented as n (%).

**Table S2-1** Summary of genotype distribution of two SNP combinations in two distinct genes by group.

| Gene combinations | Gene 1 | Gene 2 | Control group n (%) | CPP group  n (%) | p-value |
| --- | --- | --- | --- | --- | --- |
| ***IGF1R and IGF-1(1770)*** | ***IGF1R*** | ***IGF-1(1770)*** |  |  |  |
|  | AG+AA | TT | 43 (19.1) | 45 (17) | 0.121 |
|  | AG+AA | CT | 57 (25.3) | 76 (28.8) |  |
|  | AG+AA | CC | 17 (7.6) | 37 (14.0) |  |
|  | GG | TT | 25 (11.1) | 32 (12.1) |  |
|  | GG | CT | 60 (26.7) | 56 (21.2) |  |
|  | GG | CC | 23 (10.2) | 18 (6.8) |  |
|  | AG+AA | CT+CC | 74 (32.9) | 113 (42.8) | 0.090 |
|  | AG+AA | TT | 43 (19.1) | 45 (17.0) |  |
|  | GG | CT+CC | 83 (36.9) | 74 (28) |  |
|  | GG | TT | 25 (11.1) | 32 (12.1) |  |
|  |  |  |  |  |  |
| ***IGF1R and IGF-1(6093)*** | ***IGF1R*** | ***IGF-1(6093)*** |  |  |  |
|  | AG+AA | AG+AA | 36 (16.0) | 49 (18.6) | 0.168 |
|  | AG+AA | GG | 81 (36.0) | 109 (41.3) |  |
|  | GG | AG+AA | 37 (16.4) | 27 (10.2) |  |
|  | GG | GG | 71 (31.6) | 79 (29.9) |  |
|  |  |  |  |  |  |
| ***IGF2R and IGF-2(3580)*** | ***IGF2R*** | ***IGF-2(3580)*** |  |  |  |
|  | AG+AA | AG+AA | 18 (8.0) | 16 (6.1) | 0.351 |
|  | AG+AA | GG | 49 (54.2) | 64 (24.2) |  |
|  | GG | AG+AA | 36 (16.0) | 30 (11.4) |  |
|  | GG | GG | 122 (54.2) | 154 (58.3) |  |
|  |  |  |  |  |  |
| ***IGF2R and IGF-2(3123)*** | ***IGF2R*** | ***IGF-2(3123)*** |  |  |  |
|  | AG+AA | AA | 13 (5.8) | 24 (9.1) | 0.780 |
|  | AG+AA | AG | 39 (17.3) | 38 (14.4) |  |
|  | AG+AA | GG | 15 (6.7) | 18 (6.8) |  |
|  | GG | AA | 47 (20.9) | 53 (20.1) |  |
|  | GG | AG | 78 (34.7) | 92 (34.8) |  |
|  | GG | GG | 33 (14.7) | 39 (14.8) |  |
|  | AG+AA | AG+GG | 54 (24) | 56 (21.2) | 0.527 |
|  | AG+AA | AA | 13 (5.8) | 24 (9.1) |  |
|  | GG | AG+GG | 111 (49.3) | 131 (49.6) |  |
|  | GG | AA | 47 (20.9) | 53 (20.1) |  |

**Table S2-2 (continued)** Summary of genotype distribution in two SNP combinations in two distinct genes by group.

| Gene combinations | Gene 1 | Gene 2 | Control group n (%) | CPP group n (%) | p-value |
| --- | --- | --- | --- | --- | --- |
| ***IGFBP-3 and IGF-1(1770)*** | ***IGFBP-3*** | ***IGF-1(1770)*** |  |  |  |
|  | AC+CC | TT | 25 (11.1) | 30 (11.4) | 0.538 |
|  | AC+CC | CT | 54 (24.0) | 51 (19.3) |  |
|  | AC+CC | CC | 14 (6.2) | 27 (10.2) |  |
|  | AA | TT | 43 (19.1) | 47 (17.8) |  |
|  | AA | CT | 63 (28.0) | 81 (30.7) |  |
|  | AA | CC | 26 (11.6) | 28 (10.6) |  |
|  | AC+CC | CT+CC | 68 (30.2) | 78 (29.5) | 0.973 |
|  | AC+CC | TT | 25 (11.1) | 30 (11.4) |  |
|  | AA | CT+CC | 89 (39.6) | 109 (41.3) |  |
|  | AA | TT | 43 (19.1) | 47 (17.8) |  |
|  |  |  |  |  |  |
| ***IGFBP-3 and IGF-1(6093)*** | ***IGFBP-3*** | ***IGF-1 (6093)*** |  |  |  |
|  | AC+CC | AG+AA | 34 (15.1) | 35 (13.3) | 0.856 |
|  | AC+CC | GG | 59 (26.2) | 73 (27.7) |  |
|  | AA | AG+AA | 39 (17.3) | 41 (15.5) |  |
|  | AA | GG | 93 (41.3) | 115 (43.6) |  |
|  |  |  |  |  |  |
| ***IGFBP-3 and IGF-2(3580)*** | **IGFBP-3** | **IGF-2 (3580)** |  |  |  |
|  | AC+CC | AG+AA | 18 (8.0) | 22 (8.3) | 0.104 |
|  | AC+CC | GG | 75 (33.3) | 86 (32.6) |  |
|  | AA | AG+AA | 36 (16.0) | 24 (9.1) |  |
|  | AA | GG | 96 (42.7) | 132 (50.0) |  |
|  |  |  |  |  |  |
| ***IGFBP-3 and IGF-2(3123)*** | ***IGFBP-3*** | ***IGF-2 (3123)*** |  |  |  |
|  | AC+CC | AA | 24 (10.7) | 29 (11.0) | 0.982 |
|  | AC+CC | AG | 54 (24) | 59 (22.3) |  |
|  | AC+CC | GG | 15 (6.7) | 20 (7.6) |  |
|  | AA | AA | 36 (16.0) | 48 (18.2) |  |
|  | AA | AG | 63 (20.8) | 71 (26.9) |  |
|  | AA | GG | 33 (14.7) | 37 (14.0) |  |
|  | AC+CC | AG+GG | 69 (30.7) | 79 (29.9) | 0.928 |
|  | AC+CC | AA | 24 (10.7) | 29 (11.0) |  |
|  | AA | AG+GG | 96 (42.7) | 108 (40.9) |  |
|  | AA | AA | 36 (16.0) | 48 (18.2) |  |

**Table S3-1.** Associations between demographic and pathological features and SNP genotypes in control group.

| **Gene** | **Type** | **Chronological age, years** | **Bone age, years** | **BA/CA ratio** | **Age of onset, years** | **Z-scores of Height^1^** | **Z-scores of Weight^1^** | **Z-scores of BMI^1^** | **E2, pmol/l** | **FSH, U/l** | **LH, U/l** | **GH, ng/ml** | **IGF1, ng/ml** | **IGFBP-3, ng/ml** |
| --- | --- | --- | --- | --- | --- | --- | --- | --- | --- | --- | --- | --- | --- | --- |
| *IGF1R* | GG | 8.75 ± 1.44 | 8.78 ± 2.08 | 1 ± 0.18 | 7.99 ± 1.31 | 130.8 ± 8.59 | 26.48 ± 3.07 | 16.08 ± 0.67 | 35.27 ± 68.49 | 9.39 ± 7.37 | 4.43 ± 6.84 | 3.32 ± 5.42 | 263.51 ± 101.99 | 1791.15 ± 1393.69 |
|  | AG | 8.95 ± 1.61 | 9.04 ± 2.08 | 1.01 ± 0.17 | 8.17 ± 1.49 | 132.08 ± 9.54 | 26.4 ± 2.86 | 16.2 ± 0.84 | 29.38 ± 10.57 | 10.18 ± 8.01 | 7.97 ± 18.89 | 3.47 ± 5.37 | 260.25 ± 104.7 | 1898.9 ± 1441.38 |
|  | AA | 8.23 ± 1.67 | 7.91 ± 2.32 | 0.95 ± 0.17 | 7.53 ± 1.67 | 127.8 ± 10.04 | 25.23 ± 3.68 | 15.92 ± 0.53 | 28.38 ± 13.27 | 12.31 ± 9.83 | 3.54 ± 2.87 | 2.78 ± 4.17 | 230.58 ± 108.24 | 2075.76 ± 1610.28 |
|  | p-value | 0.161 | **0.033*** | 0.201 | 0.183 | 0.159 | 0.224 | 0.160 | 0.746 | 0.326 | 0.875 | 0.557 | 0.072 | 0.579 |
|  |  |  |  |  |  |  |  |  |  |  |  |  |  |  |
| *IGF-1(1770)* | TT | 8.68 ± 1.72 | 8.71 ± 2.12 | 1 ± 0.17 | 7.98 ± 1.72 | 130.46 ± 10.16 | 26.01 ± 3.37 | 16.1 ± 0.82 | 39.85 ± 85.8 | 11.67 ± 8.21 | 5.24 ± 7.76 | 3.34 ± 5.84 | 245.63 ± 101.64 | 2050.12 ± 1344.31 |
|  | CT | 8.8 ± 1.52 | 8.77 ± 2.24 | 0.99 ± 0.18 | 8.04 ± 1.42 | 131.15 ± 9.28 | 26.34 ± 3.2 | 16.12 ± 0.65 | 28.66 ± 11.44 | 9.81 ± 8.22 | 6 ± 13.29 | 3.42 ± 4.68 | 268.46 ± 105.79 | 1931.35 ± 1434.14 |
|  | CC | 8.72 ± 1.37 | 8.75 ± 1.93 | 1 ± 0.17 | 7.87 ± 0.93 | 130.54 ± 7.48 | 26.45 ± 2.41 | 16.04 ± 0.76 | 28.76 ± 10.96 | 8.37 ± 6.8 | 5.14 ± 16.85 | 2.85 ± 5.72 | 245.41 ± 102.08 | 1401.91 ± 1555.09 |
|  | p-value | 0.632 | 0.877 | 0.981 | 0.646 | 0.627 | 0.728 | 0.595 | 0.502 | 0.136 | **0.020*** | 0.451 | 0.256 | 0.111 |
|  |  |  |  |  |  |  |  |  |  |  |  |  |  |  |
| *IGF-1(6093)* | AG+GG | 8.81 ± 1.43 | 8.79 ± 2.07 | 1 ± 0.17 | 8.05 ± 1.29 | 131.2 ± 8.78 | 26.48 ± 2.9 | 16.11 ± 0.65 | 28.19 ± 10.09 | 9.99 ± 8.65 | 5.84 ± 15.76 | 3.71 ± 5.74 | 254.44 ± 106.47 | 1739.75 ± 1555.79 |
|  | GG | 8.72 ± 1.61 | 8.73 ± 2.18 | 1 ± 0.17 | 7.96 ± 1.51 | 130.66 ± 9.47 | 26.15 ± 3.21 | 16.1 ± 0.76 | 33.95 ± 58.3 | 10.18 ± 7.75 | 5.51 ± 10.84 | 3.1 ± 4.96 | 258.91 ± 103.23 | 1937.17 ± 1383.89 |
|  | p-value | 0.533 | 0.868 | 0.925 | 0.552 | 0.540 | 0.530 | 0.503 | 0.631 | 0.956 | 0.169 | 0.495 | 0.508 | 0.385 |
|  |  |  |  |  |  |  |  |  |  |  |  |  |  |  |
| *IGF2R* | GG | 8.73 ± 1.57 | 8.71 ± 2.13 | 1 ± 0.17 | 7.98 ± 1.47 | 130.71 ± 9.33 | 26.19 ± 3.12 | 16.09 ± 0.73 | 33.55 ± 57.24 | 10.28 ± 8.2 | 4.89 ± 10.2 | 3.34 ± 5.62 | 248.24 ± 98.54 | 1938.7 ± 1449.31 |
|  | AG | 8.73 ± 1.6 | 8.71 ± 2.25 | 0.99 ± 0.19 | 7.94 ± 1.43 | 130.72 ± 9.5 | 26.08 ± 3.08 | 16.1 ± 0.75 | 28.25 ± 9.6 | 9.97 ± 8.05 | 7.67 ± 18.18 | 3.04 ± 3.68 | 279.57 ± 113.46 | 1649.05 ± 1474.88 |
|  | AA | 9.25 ± 0.85 | 9.78 ± 1.56 | 1.05 ± 0.12 | 8.55 ± 0.85 | 133.85 ± 5.18 | 28.5 ± 2.53 | 16.23 ± 0.41 | 30.9 ± 12.62 | 8.22 ± 4.49 | 5.19 ± 3.81 | 4.22 ± 6.79 | 276.98 ± 123.8 | 2165.82 ± 952.3 |
|  | p-value | 0.328 | 0.298 | 0.594 | 0.280 | 0.329 | 0.135 | 0.329 | 0.872 | 0.589 | 0.446 | 0.726 | 0.198 | 0.398 |
|  |  |  |  |  |  |  |  |  |  |  |  |  |  |  |
| *IGF2R* | AG+AA | 8.8 ± 1.52 | 8.85 ± 2.19 | 1 ± 0.18 | 8.02 ± 1.38 | 131.14 ± 9.07 | 26.42 ± 3.11 | 16.11 ± 0.71 | 28.61 ± 9.99 | 9.73 ± 7.67 | 7.33 ± 16.97 | 3.2 ± 4.18 | 279.22 ± 113.92 | 1718.47 ± 1421.3 |
|  | GG | 8.73 ± 1.57 | 8.71 ± 2.13 | 1 ± 0.17 | 7.98 ± 1.47 | 130.71 ± 9.33 | 26.19 ± 3.12 | 16.09 ± 0.73 | 33.55 ± 57.24 | 10.28 ± 8.2 | 4.89 ± 10.2 | 3.34 ± 5.62 | 248.24 ± 98.54 | 1938.7 ± 1449.31 |
|  | p-value | 0.887 | 0.47 | 0.623 | 0.905 | 0.859 | 0.897 | 0.859 | 0.848 | 0.367 | 0.651 | 0.506 | 0.072 | 0.263 |
|  |  |  |  |  |  |  |  |  |  |  |  |  |  |  |
| *IGF-2(3580)* | AG+AA | 9.13 ± 1.51 | 9.33 ± 1.72 | 1.03 ± 0.13 | 8.43 ± 1.51 | 133.16 ± 8.99 | 26.36 ± 2.33 | 16.26 ± 0.85 | 42.82 ± 96.22 | 10.04 ± 7.76 | 7.29 ± 17.02 | 3.93 ± 6.95 | 274.14 ± 127.96 | 2030.85 ± 1426.67 |
|  | GG | 8.63 ± 1.55 | 8.56 ± 2.23 | 0.99 ± 0.18 | 7.85 ± 1.4 | 130.1 ± 9.22 | 26.23 ± 3.29 | 16.05 ± 0.68 | 28.66 ± 10.86 | 10.14 ± 8.14 | 5.09 ± 10.87 | 3.1 ± 4.55 | 252.2 ± 95.14 | 1823.31 ± 1446.58 |
|  | p-value | 0.141 | **0.046*** | 0.309 | 0.110 | 0.140 | 0.845 | 0.145 | 0.322 | 0.753 | 0.717 | 0.917 | 0.608 | 0.231 |
|  |  |  |  |  |  |  |  |  |  |  |  |  |  |  |
| *IGF-2(3123)* | AA | 8.77 ± 1.65 | 8.85 ± 2.41 | 1 ± 0.18 | 8.07 ± 1.65 | 131.05 ± 9.87 | 26.32 ± 3.55 | 16.13 ± 0.68 | 30.52 ± 11.97 | 9.35 ± 8.05 | 4.21 ± 5.1 | 3.63 ± 5.69 | 242.76 ± 92.23 | 1844.04 ± 1511.97 |
|  | AG | 8.73 ± 1.41 | 8.7 ± 2.03 | 0.99 ± 0.17 | 7.99 ± 1.32 | 130.72 ± 8.59 | 26.42 ± 2.99 | 16.07 ± 0.63 | 34.16 ± 65.91 | 9.85 ± 7.82 | 5.49 ± 13.19 | 3.43 ± 4.71 | 260.38 ± 105.57 | 1803.33 ± 1429.37 |
|  | GG | 8.76 ± 1.79 | 8.75 ± 2.09 | 1 ± 0.18 | 7.9 ± 1.49 | 130.84 ± 10.09 | 25.77 ± 2.85 | 16.14 ± 0.98 | 28.87 ± 10.72 | 11.71 ± 8.47 | 7.69 ± 17 | 2.56 ± 5.81 | 268.74 ± 114.05 | 2079.59 ± 1390.25 |
|  | p-value | 0.453 | 0.7 | 0.767 | 0.340 | 0.454 | 0.257 | 0.451 | 0.468 | 0.356 | 0.75 | 0.227 | 0.667 | 0.478 |
|  |  |  |  |  |  |  |  |  |  |  |  |  |  |  |
| *IGFBP-3* | AA | 8.75 ± 1.71 | 8.77 ± 2.1 | 1 ± 0.16 | 7.97 ± 1.55 | 130.81 ± 10.06 | 26.04 ± 3.14 | 16.13 ± 0.84 | 34.45 ± 62.16 | 10.68 ± 8.79 | 5.47 ± 10.42 | 3.54 ± 5.75 | 244.54 ± 106.97 | 1924.45 ± 1500.23 |
|  | AC | 8.76 ± 1.34 | 8.75 ± 2.26 | 0.99 ± 0.18 | 8.06 ± 1.34 | 130.95 ± 8.09 | 26.65 ± 3.18 | 16.07 ± 0.51 | 28.22 ± 9.73 | 9.26 ± 6.87 | 5 ± 12.08 | 3.05 ± 4.51 | 271.75 ± 96.81 | 1791.53 ± 1359.08 |
|  | CC | 8.62 ± 1.2 | 8.5 ± 1.84 | 0.99 ± 0.2 | 7.72 ± 0.71 | 130.17 ± 7.35 | 25.88 ± 1.95 | 15.98 ± 0.64 | 32.76 ± 19.04 | 9.73 ± 6.36 | 12.79 ± 31.43 | 2.15 ± 3.14 | 309.5 ± 101.52 | 1872.77 ± 1421.44 |
|  | p-value | 0.263 | 0.839 | 0.921 | 0.180 | 0.272 | 0.125 | 0.270 | 0.994 | 0.773 | 0.99 | 0.623 | 0.014* | 0.712 |
|  |  |  |  |  |  |  |  |  |  |  |  |  |  |  |
| *IGFBP-3* | AC+CC | 8.74 ± 1.32 | 8.72 ± 2.21 | 0.99 ± 0.19 | 8.02 ± 1.29 | 130.87 ± 7.98 | 26.56 ± 3.07 | 16.06 ± 0.52 | 28.66 ± 10.91 | 9.31 ± 6.78 | 5.83 ± 15.25 | 2.96 ± 4.38 | 275.81 ± 97.47 | 1800.27 ± 1358.17 |
|  | AA | 8.75 ± 1.71 | 8.77 ± 2.1 | 1 ± 0.16 | 7.97 ± 1.55 | 130.81 ± 10.06 | 26.04 ± 3.14 | 16.13 ± 0.84 | 34.45 ± 62.16 | 10.68 ± 8.79 | 5.47 ± 10.42 | 3.54 ± 5.75 | 244.54 ± 106.97 | 1924.45 ± 1500.23 |
|  | p-value | 0.616 | 0.994 | 0.774 | 0.550 | 0.625 | 0.330 | 0.625 | 0.911 | 0.514 | 0.996 | 0.33 | 0.008* | 0.433 |
|  |  |  |  |  |  |  |  |  |  |  |  |  |  |  |
| *Insulin* | TT | 8.72 ± 1.58 | 8.74 ± 2.14 | 1 ± 0.17 | 7.96 ± 1.46 | 130.65 ± 9.38 | 26.19 ± 3.16 | 16.09 ± 0.74 | 31.93 ± 49.88 | 10.3 ± 8 | 5.39 ± 10.95 | 3.33 ± 5.29 | 256.5 ± 104.2 | 1876.12 ± 1421.63 |
|  | CC | 9.16 ± 1.07 | 8.87 ± 2.17 | 0.96 ± 0.18 | 8.46 ± 1.07 | 133.38 ± 6.56 | 27.29 ± 2.13 | 16.21 ± 0.56 | 34.04 ± 9.63 | 7.48 ± 8.33 | 8.81 ± 27.24 | 2.91 ± 4.2 | 270.97 ± 104.99 | 1831.19 ± 1751.83 |
|  | p-value | 0.211 | 0.773 | 0.435 | 0.184 | 0.218 | 0.319 | 0.218 | **0.019*** | 0.11 | 0.165 | 0.948 | 0.701 | 0.741 |

^1^ Z-scores calculated with WHO Child Growth Standards for children up to 5 years old [25] and WHO 2007 reference for children older than 5 years [26].

*p<0.05.

**Table S3-2.** Associations between demographic and pathological features and SNP genotypes in CPP group.

| **Gene** | **Type** | **Chronological age, years** | **Bone age, years** | **BA/CA ratio** | **Age of onset, years** |  | **Z-scores of height^1^** |  | **Z-scores of weight^1^** |  | **Z-scores of BMI^1^** | **E2, pmol/l** | **FSH, U/l** | **LH, U/l** | **GH, ng/ml** | **IGF1, ng/ml** | **IGFBP-3, ng/ml** |
| --- | --- | --- | --- | --- | --- | --- | --- | --- | --- | --- | --- | --- | --- | --- | --- | --- | --- |
| *IGF1R* | GG | 8.51 ± 1.1 | 10.55 ± 1.26 | 1.24 ± 0.08 | 7.35 ± 0.65 |  | 129.68 ± 6.52 |  | 26.65 ± 3.16 |  | 15.95 ± 0.43 | 35.69 ± 21.5 | 17.38 ± 5.61 | 40.9 ± 36 | 3.5 ± 4.14 | 375.48 ± 116.01 | 2617.32 ± 680.12 |
|  | AG | 8.48 ± 1.07 | 10.48 ± 1.29 | 1.24 ± 0.07 | 7.32 ± 0.66 |  | 129.49 ± 6.31 |  | 26.71 ± 3.16 |  | 15.94 ± 0.4 | 31.91 ± 17.22 | 17.87 ± 6.18 | 36.48 ± 29.07 | 3.36 ± 5.25 | 366.6 ± 115.46 | 2624.13 ± 732.28 |
|  | AA | 8.48 ± 1.69 | 10.52 ± 2.11 | 1.24 ± 0.06 | 7.23 ± 1.41 |  | 129.09 ± 12 |  | 26.92 ± 4.46 |  | 16.05 ± 0.38 | 30.95 ± 11.34 | 21.08 ± 19.17 | 41.31 ± 39.34 | 4.5 ± 5.85 | 417.86 ± 120.35 | 2701.71 ± 667.23 |
|  | p-value | 0.8 | 0.456 | 0.762 | 0.818 |  | 0.728 |  | 0.600 |  | 0.389 | 0.178 | 0.909 | 0.651 | 0.345 | 0.076 | 0.701 |
|  |  |  |  |  |  |  |  |  |  |  |  |  |  |  |  |  |  |
| *IGF-1(1770)* | TT | 8.53 ± 1.35 | 10.44 ± 1.64 | 1.23 ± 0.06 | 7.31 ± 1.04 |  | 129.6 ± 8.98 |  | 26.98 ± 3.72 |  | 16 ± 0.38 | 34.25 ± 15.16 | 18.94 ± 13.01 | 40.65 ± 37.65 | 2.95 ± 4.62 | 368.09 ± 125.04 | 2598.11 ± 711.9 |
|  | CT | 8.48 ± 1.11 | 10.58 ± 1.31 | 1.25 ± 0.09 | 7.32 ± 0.64 |  | 129.51 ± 6.55 |  | 26.61 ± 3.23 |  | 15.94 ± 0.43 | 34.75 ± 22.63 | 17.52 ± 6.06 | 37.22 ± 31.25 | 3.58 ± 4.69 | 371.68 ± 113.04 | 2675.62 ± 704.22 |
|  | CC | 8.47 ± 1.04 | 10.44 ± 1.2 | 1.23 ± 0.06 | 7.34 ± 0.64 |  | 129.43 ± 6.16 |  | 26.57 ± 2.97 |  | 15.93 ± 0.39 | 28.56 ± 9.27 | 18.09 ± 5.61 | 40.11 ± 31.61 | 4.32 ± 5.72 | 398.33 ± 113.55 | 2567.6 ± 688.79 |
|  | p-value | 0.597 | 0.485 | 0.23 | 0.514 |  | 0.582 |  | 0.409 |  | 0.396 | 0.195 | 0.678 | 0.683 | 0.389 | 0.302 | 0.618 |
|  |  |  |  |  |  |  |  |  |  |  |  |  |  |  |  |  |  |
| *IGF-1(6093)* | AG+GG | 8.3 ± 1.16 | 10.29 ± 1.41 | 1.24 ± 0.08 | 7.2 ± 0.75 |  | 128.44 ± 6.85 |  | 26.26 ± 3.45 |  | 15.88 ± 0.41 | 32.23 ± 15.14 | 17.8 ± 5.72 | 40.53 ± 33.07 | 3.7 ± 5.36 | 381.6 ± 110.7 | 2704.16 ± 730.51 |
|  | GG | 8.57 ± 1.16 | 10.6 ± 1.38 | 1.24 ± 0.07 | 7.37 ± 0.78 |  | 129.96 ± 7.37 |  | 26.89 ± 3.27 |  | 15.99 ± 0.4 | 33.75 ± 19.82 | 18.15 ± 9.54 | 38.13 ± 33.35 | 3.48 ± 4.73 | 374 ± 119.51 | 2600.73 ± 690.15 |
|  | p-value | 0.086 | 0.08 | 0.68 | 0.058 |  | 0.072 |  | 0.119 |  | 0.054 | 0.738 | 0.927 | 0.456 | 0.646 | 0.656 | 0.389 |
|  |  |  |  |  |  |  |  |  |  |  |  |  |  |  |  |  |  |
| *IGF2R* | GG | 8.47 ± 1.28 | 10.5 ± 1.54 | 1.24 ± 0.08 | 7.27 ± 0.87 |  | 129.35 ± 8.02 |  | 26.62 ± 3.59 |  | 15.96 ± 0.44 | 33.97 ± 20.13 | 17.91 ± 9.43 | 38.41 ± 33.24 | 3.68 ± 5.03 | 372.87 ± 119.71 | 2659.77 ± 747.84 |
|  | AG | 8.56 ± 0.85 | 10.55 ± 0.97 | 1.23 ± 0.06 | 7.45 ± 0.47 |  | 129.93 ± 5.03 |  | 26.92 ± 2.66 |  | 15.94 ± 0.34 | 32.42 ± 15.14 | 18.47 ± 6.45 | 40.79 ± 35.24 | 3.28 ± 4.87 | 380.92 ± 111.93 | 2527.73 ± 592.75 |
|  | AA | 8.53 ± 0.9 | 10.46 ± 1.13 | 1.23 ± 0.05 | 7.39 ± 0.5 |  | 129.76 ± 5.31 |  | 26.84 ± 2.76 |  | 15.93 ± 0.35 | 28.6 ± 9.46 | 17.92 ± 6.08 | 34.46 ± 21.68 | 3.05 ± 3.36 | 398.77 ± 105.45 | 2746.03 ± 507.94 |
|  | p-value | 0.986 | 0.76 | 0.929 | 0.65 |  | 1.000 |  | 0.960 |  | 0.989 | 0.823 | 0.694 | 0.778 | 0.744 | 0.713 | 0.332 |
|  |  |  |  |  |  |  |  |  |  |  |  |  |  |  |  |  |  |
| *IGF2R* | AG+AA | 8.56 ± 0.85 | 10.54 ± 0.99 | 1.23 ± 0.06 | 7.44 ± 0.47 |  | 129.91 ± 5.04 |  | 26.91 ± 2.66 |  | 15.94 ± 0.34 | 31.8 ± 14.39 | 18.38 ± 6.36 | 39.76 ± 33.38 | 3.24 ± 4.64 | 383.82 ± 110.45 | 2563.2 ± 582.49 |
|  | GG | 8.47 ± 1.28 | 10.5 ± 1.54 | 1.24 ± 0.08 | 7.27 ± 0.87 |  | 129.35 ± 8.02 |  | 26.62 ± 3.59 |  | 15.96 ± 0.44 | 33.97 ± 20.13 | 17.91 ± 9.43 | 38.41 ± 33.24 | 3.68 ± 5.03 | 372.87 ± 119.71 | 2659.77 ± 747.84 |
|  | p-value | 0.995 | 0.459 | 0.914 | 0.397 |  | 0.999 |  | 0.775 |  | 0.886 | 0.803 | 0.422 | 0.555 | 0.745 | 0.567 | 0.48 |
|  |  |  |  |  |  |  |  |  |  |  |  |  |  |  |  |  |  |
| *IGF-2(3580)* | AG+AA | 8.27 ± 1.48 | 10.3 ± 1.91 | 1.25 ± 0.07 | 7.18 ± 1.2 |  | 127.94 ± 10.24 |  | 26.18 ± 3.95 |  | 15.92 ± 0.38 | 31.59 ± 18.13 | 18.64 ± 16.08 | 33.5 ± 20.6 | 2.99 ± 3.82 | 395.01 ± 105.01 | 2652.32 ± 743.78 |
|  | GG | 8.54 ± 1.09 | 10.56 ± 1.26 | 1.24 ± 0.08 | 7.35 ± 0.65 |  | 129.85 ± 6.42 |  | 26.82 ± 3.17 |  | 15.96 ± 0.42 | 33.68 ± 18.69 | 17.93 ± 6.01 | 39.94 ± 35.25 | 3.66 ± 5.11 | 372.21 ± 119.08 | 2625.9 ± 694.78 |
|  | p-value | 0.459 | 0.618 | 0.653 | 0.437 |  | 0.437 |  | 0.554 |  | 0.747 | 0.468 | 0.134 | 0.803 | 0.637 | 0.137 | 0.958 |
|  |  |  |  |  |  |  |  |  |  |  |  |  |  |  |  |  |  |
| *IGF-2(3123)* | AA | 8.59 ± 1.21 | 10.49 ± 1.47 | 1.22 ± 0.04 | 7.34 ± 0.74 |  | 130.16 ± 7.14 |  | 26.98 ± 3.49 |  | 16 ± 0.46 | 34.73 ± 17.35 | 17.74 ± 6.06 | 39.52 ± 32.63 | 2.99 ± 4.23 | 377.15 ± 115.92 | 2615.54 ± 692.44 |
|  | AG | 8.49 ± 1.19 | 10.52 ± 1.43 | 1.24 ± 0.08 | 7.31 ± 0.84 |  | 129.43 ± 7.75 |  | 26.68 ± 3.33 |  | 15.96 ± 0.39 | 34.11 ± 20.87 | 18.1 ± 10.57 | 38.56 ± 35.48 | 4.07 ± 5.31 | 368.83 ± 118.36 | 2621.01 ± 689.24 |
|  | GG | 8.37 ± 1.04 | 10.51 ± 1.21 | 1.26 ± 0.1 | 7.31 ± 0.66 |  | 128.85 ± 6.15 |  | 26.42 ± 3.13 |  | 15.89 ± 0.38 | 29.59 ± 13.8 | 18.35 ± 6.36 | 38.48 ± 28.96 | 3.11 ± 4.76 | 391.65 ± 115.24 | 2672.39 ± 753.81 |
|  | p-value | 0.365 | 0.936 | 0.240 | 0.734 |  | 0.361 |  | 0.491 |  | 0.354 | 0.15 | 0.588 | 0.745 | 0.636 | 0.352 | 0.888 |
|  |  |  |  |  |  |  |  |  |  |  |  |  |  |  |  |  |  |
| *IGFBP-3* | AA | 8.56 ± 1.07 | 10.59 ± 1.25 | 1.24 ± 0.07 | 7.35 ± 0.65 |  | 129.94 ± 6.35 |  | 26.91 ± 3.18 |  | 15.97 ± 0.41 | 34.36 ± 21.55 | 17.51 ± 5.96 | 38.09 ± 33.19 | 3.02 ± 4.11 | 364.42 ± 115.96 | 2688.77 ± 643.84 |
|  | AC | 8.36 ± 1.32 | 10.38 ± 1.63 | 1.24 ± 0.09 | 7.25 ± 0.98 |  | 128.64 ± 8.66 |  | 26.36 ± 3.63 |  | 15.93 ± 0.4 | 32.04 ± 13.58 | 18.75 ± 11.93 | 37.2 ± 27.83 | 4.17 ± 5.58 | 391.37 ± 116.68 | 2525.48 ± 780.96 |
|  | CC | 8.66 ± 1.03 | 10.53 ± 1.25 | 1.22 ± 0.05 | 7.47 ± 0.5 |  | 130.56 ± 6.16 |  | 26.82 ± 2.86 |  | 16 ± 0.44 | 30.37 ± 10.03 | 19.28 ± 7.43 | 56.53 ± 55.59 | 5.11 ± 7.3 | 404.36 ± 119.87 | 2675.8 ± 742.64 |
|  | p-value | 0.543 | 0.768 | 0.735 | 0.633 |  | 0.565 |  | 0.586 |  | 0.733 | 0.952 | 0.663 | 0.468 | 0.253 | 0.112 | 0.148 |
|  |  |  |  |  |  |  |  |  |  |  |  |  |  |  |  |  |  |
| *IGFBP-3* | AC+CC | 8.4 ± 1.28 | 10.4 ± 1.58 | 1.24 ± 0.08 | 7.28 ± 0.93 |  | 128.91 ± 8.36 |  | 26.42 ± 3.53 |  | 15.94 ± 0.41 | 31.8 ± 13.12 | 18.82 ± 11.38 | 39.88 ± 33.4 | 4.31 ± 5.81 | 393.17 ± 116.65 | 2546.35 ± 774.14 |
|  | AA | 8.56 ± 1.07 | 10.59 ± 1.25 | 1.24 ± 0.07 | 7.35 ± 0.65 |  | 129.94 ± 6.35 |  | 26.91 ± 3.18 |  | 15.97 ± 0.41 | 34.36 ± 21.55 | 17.51 ± 5.96 | 38.09 ± 33.19 | 3.02 ± 4.11 | 364.42 ± 115.96 | 2688.77 ± 643.84 |
|  | p-value | 0.347 | 0.473 | 0.634 | 0.900 |  | 0.362 |  | 0.311 |  | 0.507 | 0.871 | 0.462 | 0.354 | 0.130 | 0.038* | 0.071 |
|  |  |  |  |  |  |  |  |  |  |  |  |  |  |  |  |  |  |
| *Insulin* | TT | 8.49 ± 1.17 | 10.5 ± 1.41 | 1.24 ± 0.08 | 7.32 ± 0.79 |  | 129.5 ± 7.32 |  | 26.7 ± 3.34 |  | 15.96 ± 0.41 | 33.51 ± 18.79 | 18.02 ± 8.73 | 39.18 ± 33.41 | 3.53 ± 4.97 | 377.41 ± 116.88 | 2627.44 ± 689.06 |
|  | CC | 8.55 ± 1.02 | 10.64 ± 1.15 | 1.25 ± 0.06 | 7.39 ± 0.55 |  | 129.88 ± 6.05 |  | 26.94 ± 3.23 |  | 15.95 ± 0.42 | 29.74 ± 14.35 | 18.56 ± 6.24 | 32.36 ± 30.1 | 3.84 ± 3.67 | 354.35 ± 119.15 | 2685.27 ± 936.48 |
|  | p-value | 0.924 | 0.861 | 0.121 | 0.865 |  | 0.872 |  | 0.952 |  | 0.832 | 0.486 | 0.581 | 0.246 | 0.181 | 0.416 | 0.427 |

^1^ Z-scores calculated with WHO Child Growth Standards for children up to 5 years old [25] and WHO 2007 reference for children older than 5 years [26].

*p<0.05.

**S4-1.** Comparison of associations between demographic and pathological features with two SNP genotype combinations in the control group.

| **Gene 1** | **Gene 2** | **n** | **Chronological age, years** | **Bone age, years** | **BA/CA ratio** | **Onset age, years** | **Z-scores of height^1^** | **Z-scores of weight^1^** | **Z-scores of BMI^1^** | **E2, pmol/l** | **FSH, U/l** | **LH, U/l** | **GH, ng/ml** | **IGF1, ng/ml** | **IGFBP-3, ng/ml** |
| --- | --- | --- | --- | --- | --- | --- | --- | --- | --- | --- | --- | --- | --- | --- | --- |
| *IGF-1(1770) (TT)* | *IGF1R (GG)* |  |  |  |  |  |  |  |  |  |  |  |  |  |  |
| + | + | 25 | 8.83 ± 1.47 | 8.88 ± 1.92 | 1.01 ± 0.19 | 8.04 ± 1.36 | 131.38 ± 8.78 | 26.43 ± 2.85 | 16.12 ± 0.79 | 58.7 ± 140.75 | 9.77 ± 6.89 | 4.16 ± 5.41 | 3.96 ± 4.78 | 253.84 ± 101.51 | 1889.73 ± 1362.63 |
| + | - | 43 | 8.59 ± 1.86 | 8.6 ± 2.24 | 1 ± 0.16 | 7.89 ± 1.86 | 130.62 ± 8.58 | 26.49 ± 3.15 | 16.07 ± 0.64 | 28.89 ± 9.46 | 12.78 ± 8.78 | 5.88 ± 8.84 | 2.99 ± 6.4 | 240.85 ± 102.6 | 2143.37 ± 1340.76 |
| - | + | 83 | 8.72 ± 1.44 | 8.75 ± 2.14 | 1 ± 0.17 | 7.95 ± 1.27 | 129.93 ± 10.95 | 25.76 ± 3.66 | 16.09 ± 0.85 | 28.21 ± 10.26 | 9.28 ± 7.55 | 4.51 ± 7.24 | 3.12 ± 5.61 | 266.43 ± 102.56 | 1761.46 ± 1409.7 |
| - | - | 74 | 8.84 ± 1.53 | 8.78 ± 2.19 | 0.99 ± 0.17 | 8.13 ± 1.47 | 131.41 ± 9.15 | 26.22 ± 2.82 | 16.13 ± 0.72 | 29.22 ± 12.39 | 9.62 ± 8.3 | 7.21 ± 19.23 | 3.45 ± 4.12 | 258.29 ± 108.24 | 1835.72 ± 1561.88 |
| p-value |  |  | 0.886 | 0.975 | 0.881 | 0.93 | 0.885 | 0.711 | 0.871 | 0.617 | 0.133 | 0.151 | 0.198 | 0.608 | 0.657 |
|  |  |  |  |  |  |  |  |  |  |  |  |  |  |  |  |
| *IGF-1(6093) (GG)* | *IGF1R (GG)* | n |  |  |  |  |  |  |  |  |  |  |  |  |  |
| + | + | 71 | 8.84 ± 1.46 | 8.71 ± 2.12 | 0.99 ± 0.19 | 8.24 ± 1.15 | 131.4 ± 8.38 | 26.56 ± 2.93 | 16.12 ± 0.77 | 38.9 ± 84.13 | 9.34 ± 6.43 | 5.09 ± 7.98 | 3.33 ± 4.71 | 267.37 ± 97.59 | 1889.37 ± 1351.79 |
| + | - | 81 | 8.6 ± 1.73 | 8.74 ± 2.25 | 1.01 ± 0.16 | 7.88 ± 1.7 | 129.65 ± 8.97 | 26.32 ± 3.34 | 16 ± 0.42 | 29.56 ± 11.89 | 10.91 ± 8.71 | 5.89 ± 12.88 | 2.89 ± 5.19 | 251.5 ± 107.99 | 1979.08 ± 1418.5 |
| - | + | 37 | 8.56 ± 1.41 | 8.92 ± 2.03 | 1.04 ± 0.15 | 7.86 ± 1.41 | 130.01 ± 10.34 | 25.8 ± 3.42 | 16.08 ± 0.75 | 28.31 ± 10.19 | 9.51 ± 9.01 | 3.17 ± 3.56 | 3.29 ± 6.65 | 256.11 ± 110.95 | 1602.68 ± 1471.22 |
| - | - | 36 | 9.07 ± 1.43 | 8.67 ± 2.12 | 0.95 ± 0.18 | 8.06 ± 1.27 | 132.8 ± 8.4 | 26.66 ± 2.31 | 16.22 ± 0.81 | 28.07 ± 10.12 | 10.48 ± 8.37 | 8.58 ± 21.96 | 4.15 ± 4.68 | 252.74 ± 103.2 | 1880.63 ± 1647.03 |
| p-value |  |  | 0.64 | 0.9 | 0.131 | 0.70 | 0.640 | 0.681 | 0.650 | 0.957 | 0.748 | 0.548 | 0.312 | 0.561 | 0.496 |
|  |  |  |  |  |  |  |  |  |  |  |  |  |  |  |  |
| *IGF-2(3580) (GG)* | *IGF2R (GG)* |  |  |  |  |  |  |  |  |  |  |  |  |  |  |
| + | + | 122 | 8.59 ± 1.55 | 8.53 ± 2.18 | 0.99 ± 0.18 | 8.3 ± 1.39 | 129.87 ± 9.2 | 26.11 ± 3.24 | 16.03 ± 0.67 | 28.85 ± 11.43 | 10.17 ± 8.32 | 4.67 ± 10.32 | 3.27 ± 5.01 | 249.71 ± 93.75 | 1856.47 ± 1465.74 |
| + | - | 49 | 8.72 ± 1.58 | 8.65 ± 2.39 | 0.98 ± 0.19 | 7.92 ± 1.38 | 133.52 ± 9.34 | 26.5 ± 2.6 | 16.3 ± 0.88 | 28.2 ± 9.41 | 10.06 ± 7.76 | 6.14 ± 12.18 | 2.66 ± 3.13 | 258.38 ± 99.23 | 1740.75 ± 1409.19 |
| - | + | 36 | 9.19 ± 1.58 | 9.31 ± 1.85 | 1.02 ± 0.14 | 8.49 ± 1.58 | 130.67 ± 9.33 | 26.52 ± 3.44 | 16.09 ± 0.69 | 49.37 ± 117.57 | 10.65 ± 7.91 | 5.65 ± 9.89 | 3.56 ± 7.41 | 243.24 ± 114.64 | 2217.37 ± 1375.3 |
| - | - | 18 | 9 ± 1.39 | 9.39 ± 1.46 | 1.05 ± 0.13 | 7.83 ± 1.41 | 132.42 ± 8.45 | 26.09 ± 1.73 | 16.18 ± 0.78 | 29.73 ± 11.66 | 8.82 ± 7.53 | 10.58 ± 26.16 | 4.67 ± 6.08 | 335.94 ± 133.88 | 1657.8 ± 1493.44 |
| p-value |  |  | 0.401 | 0.213 | 0.706 | 0.35 | 0.394 | 0.834 | 0.402 | 0.778 | 0.746 | 0.695 | 0.736 | 0.052 | 0.325 |
|  |  |  |  |  |  |  |  |  |  |  |  |  |  |  |  |
| *IGF-2(3123) (AA)* | *IGF2R (GG)* |  |  |  |  |  |  |  |  |  |  |  |  |  |  |
| + | + | 47 | 8.76 ± 1.7 | 8.62 ± 2.43 | 0.98 ± 0.19 | 8 ± 1.36 | 130.98 ± 10.2 | 25.77 ± 3.28 | 16.13 ± 0.75 | 31.81 ± 12.62 | 8.42 ± 7.47 | 3.63 ± 5.11 | 4.13 ± 6.32 | 237.63 ± 96.23 | 1865.77 ± 1609.92 |
| + | - | 13 | 8.82 ± 1.51 | 9.69 ± 2.25 | 1.08 ± 0.12 | 8.12 ± 1.51 | 130.59 ± 8.98 | 26.35 ± 3.06 | 16.08 ± 0.73 | 25.83 ± 8.01 | 12.74 ± 9.44 | 6.28 ± 4.68 | 1.81 ± 1.27 | 261.27 ± 76.5 | 1765.49 ± 1138.37 |
| - | + | 111 | 8.72 ± 1.52 | 8.74 ± 2 | 1 ± 0.16 | 7.94 ± 1.37 | 131.32 ± 8.98 | 27.94 ± 3.94 | 16.13 ± 0.36 | 34.3 ± 67.97 | 11.07 ± 8.4 | 5.43 ± 11.69 | 3 ± 5.29 | 252.73 ± 99.6 | 1969.58 ± 1382.35 |
| - | - | 54 | 8.79 ± 1.54 | 8.65 ± 2.15 | 0.98 ± 0.19 | 8.06 ± 1.7 | 131.1 ± 9.18 | 25.98 ± 2.72 | 16.11 ± 0.78 | 29.28 ± 10.36 | 9.01 ± 7.09 | 7.59 ± 18.8 | 3.54 ± 4.56 | 283.54 ± 121.4 | 1707.15 ± 1490.47 |
| p-value |  |  | 0.398 | 0.196 | 0.152 | 0.34 | 0.114 | 0.012* | 0.114 | 0.227 | 0.101 | 0.062 | 0.358 | 0.294 | 0.673 |
|  |  |  |  |  |  |  |  |  |  |  |  |  |  |  |  |
| *IGF-1(1770) (TT)* | *IGFBP-3 (AA)* |  |  |  |  |  |  |  |  |  |  |  |  |  |  |
| + | + | 43 | 8.77 ± 1.92 | 8.84 ± 1.94 | 1.01 ± 0.14 | 8.1 ± 1.27 | 131.04 ± 11.26 | 25.62 ± 3.24 | 16.18 ± 0.99 | 45.67 ± 107.6 | 11.7 ± 8.77 | 5.53 ± 8.96 | 3.9 ± 6.43 | 241.26 ± 108.45 | 2159.44 ± 1478.24 |
| + | - | 25 | 8.52 ± 1.34 | 8.48 ± 2.42 | 0.99 ± 0.21 | 7.82 ± 1.34 | 130.7 ± 9.49 | 26.23 ± 3.09 | 16.1 ± 0.76 | 29.86 ± 11.17 | 11.63 ± 7.33 | 4.75 ± 5.21 | 2.38 ± 4.61 | 253.13 ± 90.37 | 1862.09 ± 1078.29 |
| - | + | 89 | 8.74 ± 1.61 | 8.74 ± 2.18 | 1 ± 0.17 | 7.92 ± 1.34 | 129.47 ± 8.06 | 26.59 ± 3.56 | 15.97 ± 0.4 | 29.03 ± 11.64 | 10.19 ± 8.81 | 5.44 ± 11.11 | 3.36 ± 5.42 | 246.12 ± 106.83 | 1810.91 ± 1505.82 |
| - | - | 68 | 8.83 ± 1.31 | 8.81 ± 2.15 | 0.99 ± 0.17 | 8.07 ± 1.92 | 131.38 ± 7.95 | 26.56 ± 2.87 | 16.1 ± 0.56 | 28.22 ± 10.86 | 8.46 ± 6.42 | 6.23 ± 17.58 | 3.17 ± 4.3 | 284.15 ± 99.28 | 1777.54 ± 1454.12 |
| p-value |  |  | 0.848 | 0.965 | 0.994 | 0.87 | 0.852 | 0.446 | 0.839 | 0.694 | 0.209 | 0.382 | 0.132 | **0.038*** | 0.534 |
|  |  |  |  |  |  |  |  |  |  |  |  |  |  |  |  |
| *IGF-1(6093) (GG)* | *IGFBP-3 (AA)* |  |  |  |  |  |  |  |  |  |  |  |  |  |  |
| + | + | 93 | 8.72 ± 1.73 | 8.74 ± 2.08 | 1 ± 0.16 | 8.1 ± 1.15 | 130.67 ± 10.04 | 25.84 ± 3.05 | 16.12 ± 0.88 | 37.2 ± 73.7 | 10.59 ± 8.28 | 5.43 ± 8.87 | 3.46 ± 5.34 | 252.53 ± 108.05 | 2010.8 ± 1464.52 |
| + | - | 59 | 8.71 ± 1.41 | 8.71 ± 2.35 | 0.99 ± 0.19 | 7.97 ± 1.37 | 131.16 ± 10.21 | 26.51 ± 3.33 | 16.15 ± 0.73 | 28.74 ± 11.54 | 9.53 ± 6.83 | 5.64 ± 13.46 | 2.52 ± 4.28 | 268.98 ± 95.17 | 1821.12 ± 1249.59 |
| - | + | 39 | 8.83 ± 1.65 | 8.85 ± 2.16 | 1 ± 0.16 | 8 ± 1.42 | 130.64 ± 8.57 | 26.63 ± 3.42 | 16.06 ± 0.52 | 27.89 ± 10.37 | 10.9 ± 10 | 5.55 ± 13.57 | 3.71 ± 6.7 | 225.48 ± 103.2 | 1718.53 ± 1582.54 |
| - | - | 34 | 8.8 ± 1.15 | 8.74 ± 1.99 | 0.99 ± 0.18 | 7.96 ± 1.61 | 131.26 ± 6.94 | 26.45 ± 2.34 | 16.06 ± 0.54 | 28.53 ± 9.9 | 8.94 ± 6.78 | 6.17 ± 18.15 | 3.71 ± 4.5 | 287.67 ± 101.68 | 1764.09 ± 1547.92 |
| p-value |  |  | 0.691 | 0.984 | 0.989 | 0.71 | 0.705 | 0.300 | 0.659 | 0.726 | 0.935 | 0.575 | 0.235 | **0.013*** | 0.511 |
|  |  |  |  |  |  |  |  |  |  |  |  |  |  |  |  |
| *IGF-2(3580) (GG)* | *IGFBP-3 (AA)* |  |  |  |  |  |  |  |  |  |  |  |  |  |  |
| + | + | 96 | 8.61 ± 1.73 | 8.54 ± 2.21 | 0.99 ± 0.17 | 8.42 ± 1.33 | 129.94 ± 10.16 | 25.96 ± 3.4 | 16.07 ± 0.8 | 28.55 ± 10.51 | 10.58 ± 9.11 | 5.13 ± 10.12 | 3.56 ± 5.16 | 240 ± 96.22 | 1805.07 ± 1531.63 |
| + | - | 75 | 8.65 ± 1.31 | 8.6 ± 2.28 | 0.99 ± 0.19 | 7.93 ± 1.27 | 133.14 ± 9.52 | 26.29 ± 2.18 | 16.27 ± 0.93 | 28.8 ± 11.38 | 9.58 ± 6.71 | 5.05 ± 11.83 | 2.51 ± 3.56 | 267.8 ± 92.02 | 1846.67 ± 1339.68 |
| - | + | 36 | 9.13 ± 1.61 | 9.39 ± 1.64 | 1.03 ± 0.13 | 8.43 ± 1.61 | 130.31 ± 7.91 | 26.57 ± 3.15 | 16.02 ± 0.49 | 50.17 ± 117.54 | 10.95 ± 7.98 | 6.38 ± 11.28 | 3.49 ± 7.16 | 256.63 ± 132.25 | 2242.8 ± 1383.2 |
| - | - | 18 | 9.12 ± 1.33 | 9.22 ± 1.9 | 1.01 ± 0.14 | 7.8 ± 1.5 | 133.19 ± 8.08 | 26.52 ± 2.71 | 16.23 ± 0.66 | 28.12 ± 8.97 | 8.22 ± 7.17 | 9.12 ± 25.21 | 4.83 ± 6.62 | 309.16 ± 114.41 | 1606.94 ± 1456.42 |
| p-value |  |  | 0.461 | 0.241 | 0.75 | 0.37 | 0.461 | 0.742 | 0.470 | 0.786 | 0.686 | 0.565 | 0.23 | **0.036*** | 0.303 |
|  |  |  |  |  |  |  |  |  |  |  |  |  |  |  |  |
| *IGF-2(3123) (AA)* | *IGFBP-3 (AA)* |  |  |  |  |  |  |  |  |  |  |  |  |  |  |
| + | + | 36 | 8.68 ± 1.73 | 8.86 ± 2.26 | 1.02 ± 0.17 | 7.96 ± 1.2 | 130.52 ± 10.31 | 26.07 ± 3.64 | 16.11 ± 0.74 | 30.31 ± 12.19 | 10.28 ± 8.78 | 4.61 ± 5.83 | 4.32 ± 6.84 | 227.31 ± 100.55 | 1827.22 ± 1520.97 |
| + | - | 24 | 8.91 ± 1.53 | 8.83 ± 2.68 | 0.98 ± 0.2 | 8.21 ± 1.53 | 130.93 ± 10.01 | 26.02 ± 2.95 | 16.13 ± 0.88 | 30.83 ± 11.88 | 7.97 ± 6.76 | 3.6 ± 3.8 | 2.59 ± 3.16 | 265.93 ± 74.18 | 1869.27 ± 1530.63 |
| - | + | 96 | 8.78 ± 1.7 | 8.73 ± 2.05 | 1 ± 0.16 | 7.96 ± 1.48 | 131.86 ± 9.34 | 26.71 ± 3.46 | 16.17 ± 0.59 | 36 ± 72.56 | 10.83 ± 8.83 | 5.79 ± 11.7 | 3.24 ± 5.29 | 251 ± 109.08 | 1960.91 ± 1498.79 |
| - | - | 69 | 8.69 ± 1.24 | 8.68 ± 2.05 | 0.99 ± 0.18 | 7.98 ± 1.73 | 130.52 ± 7.5 | 26.52 ± 2.96 | 16.03 ± 0.5 | 27.9 ± 10.52 | 9.78 ± 6.78 | 6.61 ± 17.53 | 3.08 ± 4.74 | 279.24 ± 104.62 | 1776.27 ± 1304.21 |
| p-value |  |  | 0.723 | 0.868 | 0.865 | 0.633 | 0.722 | 0.609 | 0.731 | 0.657 | 0.614 | 0.993 | 0.399 | **0.044*** | 0.757 |

^1^ Z-scores calculated with WHO Child Growth Standards for children up to 5 years old [25] and WHO 2007 reference for children older than 5 years [26].

*p<0.05.

**Table S4-2**. Associations between demographic and pathological features and two SNP genotype combinations in the CPP group.

| **Gene 1** | **Gene 2** | **n** | **Chronological age, years** | **Bone age, years** | **BA/CA ratio** | **Age of onset, years** | **Z-scores of height^1^** | **Z-scores of Weight^1^** | **Z-scores of BMI^1^** | **E2, pmol/l** | **FSH, U/l** | **LH, U/l** | **GH, ng/ml** | **IGF1, ng/ml** | **IGFBP-3, ng/ml** | | |
| --- | --- | --- | --- | --- | --- | --- | --- | --- | --- | --- | --- | --- | --- | --- | --- | --- | --- |
| *IGF-1(1770) (TT)* | *IGF1R (GG)* |  |  |  |  |  |  |  |  |  |  |  |  |  |  | | |
| + | + | 32 | 8.63 ± 1.06 | 10.53 ± 1.27 | 1.22 ± 0.05 | 7.33 ± 0.63 | 130.39 ± 6.29 | 27.25 ± 3.27 | 16 ± 0.41 | 35.24 ± 15.63 | 17.95 ± 5.74 | 40.91 ± 37.91 | 3.39 ± 4.88 | 363.25 ± 127.48 | 2555.47 ± 694.21 | | |
| + | - | 45 | 8.45 ± 1.52 | 10.38 ± 1.87 | 1.23 ± 0.07 | 7.23 ± 1.25 | 129.37 ± 6.63 | 26.38 ± 3.1 | 15.93 ± 0.44 | 33.55 ± 14.96 | 19.65 ± 16.37 | 40.47 ± 37.89 | 2.63 ± 4.46 | 371.54 ± 124.6 | 2628.43 ± 730.47 | | |
| - | + | 74 | 8.46 ± 1.12 | 10.55 ± 1.26 | 1.25 ± 0.09 | 7.32 ± 0.66 | 129.03 ± 10.52 | 26.78 ± 4.04 | 16 ± 0.36 | 35.88 ± 23.69 | 17.14 ± 5.57 | 40.9 ± 35.41 | 3.54 ± 3.81 | 380.77 ± 111.19 | 2644.07 ± 676.96 | | |
| - | - | 113 | 8.49 ± 1.07 | 10.53 ± 1.3 | 1.24 ± 0.07 | 7.42 ± 0.62 | 129.56 ± 6.31 | 26.74 ± 3.19 | 15.94 ± 0.41 | 30.99 ± 16.68 | 18.04 ± 6.14 | 36.21 ± 28.3 | 3.96 ± 5.67 | 378.69 ± 115.53 | 2643.7 ± 717.05 | | |
| p-value |  |  | 0.756 | 0.999 | 0.459 | 0.69 | 0.735 | 0.478 | 0.564 | 0.131 | 0.834 | 0.808 | 0.435 | 0.92 | 0.834 | | |
|  |  |  |  |  |  |  |  |  |  |  |  |  |  |  |  | | |
| *IGF-1(6093) (GG)* | *IGF1R (GG)* |  |  |  |  |  |  |  |  |  |  |  |  |  |  | | |
| + | + | 79 | 8.63 ± 1.05 | 10.67 ± 1.14 | 1.24 ± 0.07 | 7.25 ± 0.71 | 130.37 ± 6.25 | 26.93 ± 3.04 | 15.99 ± 0.44 | 36.8 ± 23.18 | 17.31 ± 5.73 | 40.71 ± 37.83 | 3.59 ± 4.3 | 374.01 ± 120.43 | 2594.66 ± 682.41 | | |
| + | - | 109 | 8.53 ± 1.24 | 10.55 ± 1.53 | 1.24 ± 0.07 | 7.33 ± 0.91 | 127.65 ± 6.96 | 25.86 ± 3.41 | 15.83 ± 0.39 | 31.54 ± 16.74 | 18.76 ± 11.53 | 36.26 ± 29.73 | 3.41 ± 5.03 | 373.99 ± 119.39 | 2605.14 ± 698.81 | | |
| - | + | 27 | 8.17 ± 1.19 | 10.19 ± 1.52 | 1.25 ± 0.11 | 7.12 ± 0.81 | 129.65 ± 8.1 | 26.87 ± 3.43 | 15.98 ± 0.38 | 32.43 ± 15.52 | 17.59 ± 5.35 | 41.46 ± 30.69 | 3.23 ± 3.68 | 379.79 ± 103.99 | 2683.65 ± 681.81 | | |
| - | - | 49 | 8.38 ± 1.16 | 10.35 ± 1.36 | 1.24 ± 0.06 | 7.43 ± 0.56 | 128.88 ± 6.82 | 26.48 ± 3.48 | 15.9 ± 0.42 | 32.11 ± 15.09 | 17.92 ± 5.97 | 40.02 ± 34.61 | 3.97 ± 6.11 | 382.59 ± 115.27 | 2715.47 ± 762.66 | | |
| p-value |  |  | 0.351 | 0.373 | 0.863 | 0.23 | 0.302 | 0.396 | 0.244 | 0.257 | 0.985 | 0.663 | 0.695 | 0.968 | 0.816 | | |
|  |  |  |  |  |  |  |  |  |  |  |  |  |  |  |  | | |
| *IGF-2(3580) (GG)* | *IGF2R (GG)* |  |  |  |  |  |  |  |  |  |  |  |  |  |  | | |
| + | + | 154 | 8.54 ± 1.18 | 10.57 ± 1.36 | 1.24 ± 0.08 | 7.41 ± 0.51 | 129.85 ± 6.96 | 26.8 ± 3.39 | 15.97 ± 0.45 | 34.34 ± 19.9 | 17.47 ± 5.68 | 39.44 ± 34.81 | 3.77 ± 5.23 | 369.13 ± 120.02 | 2636.11 ± 739.66 | | |
| + | - | 64 | 8.55 ± 0.84 | 10.52 ± 0.98 | 1.23 ± 0.06 | 7.45 ± 0.47 | 126.78 ± 11.95 | 25.72 ± 4.39 | 15.9 ± 0.38 | 32.07 ± 15.44 | 19.02 ± 6.65 | 41.15 ± 36.54 | 3.4 ± 4.83 | 379.63 ± 117.38 | 2601.36 ± 577.2 | | |
| - | + | 30 | 8.1 ± 1.69 | 10.13 ± 2.22 | 1.25 ± 0.08 | 7.06 ± 1.43 | 129.85 ± 4.96 | 26.88 ± 2.62 | 15.94 ± 0.34 | 32.06 ± 21.53 | 20.16 ± 19.62 | 33.13 ± 23.31 | 3.2 ± 3.84 | 392.06 ± 118.22 | 2781.26 ± 790.29 | | |
| - | - | 16 | 8.59 ± 0.94 | 10.63 ± 1.09 | 1.24 ± 0.06 | 7.31 ± 0.71 | 130.11 ± 5.54 | 27.04 ± 2.9 | 15.96 ± 0.37 | 30.71 ± 9.42 | 15.79 ± 4.28 | 34.21 ± 14.87 | 2.62 ± 3.88 | 400.56 ± 77.53 | 2410.57 ± 597.14 | | |
|  |  |  | 0.666 | 0.676 | 0.974 | 0.71 | 0.685 | 0.772 | 0.921 | 0.735 | 0.264 | 0.82 | 0.91 | 0.478 | 0.487 | | |
|  |  |  |  |  |  |  |  |  |  |  |  |  |  |  |  | | |
| *IGF-2(3123) (AA)* | *IGF2R (GG)* |  |  |  |  |  |  |  |  |  |  |  |  |  |  | | |
| + | + | 53 | 8.56 ± 1.35 | 10.45 ± 1.62 | 1.22 ± 0.04 | 7.41 ± 0.48 | 130.03 ± 7.96 | 26.87 ± 3.83 | 16.01 ± 0.5 | 34.93 ± 18.67 | 18 ± 6.04 | 40.09 ± 33.45 | 3.2 ± 4.76 | 378.69 ± 119.85 | 2632.85 ± 716.36 | | |
| + | - | 24 | 8.65 ± 0.85 | 10.58 ± 1.06 | 1.22 ± 0.04 | 7.51 ± 0.47 | 129.08 ± 8.06 | 26.52 ± 3.5 | 15.94 ± 0.41 | 34.3 ± 14.37 | 17.16 ± 6.18 | 38.25 ± 31.41 | 2.53 ± 2.75 | 373.74 ± 109.14 | 2577.32 ± 649.55 | | |
| - | + | 131 | 8.43 ± 1.25 | 10.52 ± 1.51 | 1.25 ± 0.09 | 7.27 ± 0.89 | 130.44 ± 5.03 | 27.19 ± 2.68 | 15.98 ± 0.34 | 33.59 ± 20.75 | 17.87 ± 10.52 | 37.74 ± 33.26 | 3.87 ± 5.13 | 370.51 ± 120.03 | 2670.66 ± 762.63 | | |
| - | - | 56 | 8.52 ± 0.86 | 10.52 ± 0.97 | 1.24 ± 0.06 | 7.26 ± 0.83 | 129.68 ± 5.08 | 26.79 ± 2.67 | 15.93 ± 0.34 | 30.73 ± 14.4 | 18.9 ± 6.42 | 40.41 ± 34.45 | 3.55 ± 5.24 | 388.13 ± 111.71 | 2557.15 ± 557.48 | | |
| p-value |  |  | 0.753 | 0.881 | 0.425 | 0.63 | 0.645 | 0.736 | 0.645 | 0.6 | 0.4 | 0.906 | 0.981 | 0.856 | 0.867 | | |
|  |  |  |  |  |  |  |  |  |  |  |  |  |  |  |  | | |
| *IGF-1(1770) (TT)* | *IGFBP-3 (AA)* |  |  |  |  |  |  |  |  |  |  |  |  |  |  | | |
| + | + | 47 | 8.63 ± 1.05 | 10.57 ± 1.28 | 1.23 ± 0.04 | 7.32 ± 0.65 | 130.37 ± 6.16 | 27.23 ± 3.14 | 15.99 ± 0.38 | 34.06 ± 16.37 | 17.99 ± 5.8 | 42.1 ± 39.84 | 2.88 ± 4.61 | 356.11 ± 131.64 | 2712.71 ± 676.31 | | |
| + | - | 30 | 8.37 ± 1.73 | 10.23 ± 2.1 | 1.22 ± 0.08 | 7.16 ± 1.43 | 129.76 ± 6.45 | 26.77 ± 3.2 | 15.96 ± 0.42 | 34.55 ± 13.31 | 20.42 ± 19.66 | 38.37 ± 34.48 | 3.05 ± 4.73 | 386.87 ± 113.51 | 2418.56 ± 740.28 | | |
| - | + | 109 | 8.52 ± 1.09 | 10.6 ± 1.24 | 1.25 ± 0.08 | 7.33 ± 0.64 | 128.39 ± 12.19 | 26.58 ± 4.52 | 16.01 ± 0.38 | 34.49 ± 23.49 | 17.31 ± 6.05 | 36.36 ± 29.92 | 3.08 ± 3.89 | 368.01 ± 108.97 | 2678.44 ± 632.26 | | |
| - | - | 78 | 8.42 ± 1.08 | 10.46 ± 1.34 | 1.25 ± 0.09 | 7.4 ± 0.68 | 129.11 ± 6.4 | 26.36 ± 3.09 | 15.91 ± 0.42 | 30.75 ± 12.97 | 18.21 ± 5.75 | 40.46 ± 33.18 | 4.79 ± 6.14 | 395.6 ± 118.46 | 2595.5 ± 785.86 | | |
| p-value |  |  | 0.596 | 0.908 | 0.322 | 0.6 | 0.599 | 0.427 | 0.472 | 0.367 | 0.674 | 0.808 | 0.135 | 0.207 | 0.257 | | |
|  |  |  |  |  |  |  |  |  |  |  |  |  |  |  |  | | |
| *IGF-1(6093) (GG)* | *IGFBP-3 (AA)* |  |  |  |  |  |  |  |  |  |  |  |  |  |  | | |
| + | + | 115 | 8.64 ± 0.99 | 10.72 ± 1.11 | 1.24 ± 0.07 | 7.33 ± 0.69 | 130.46 ± 5.86 | 27.12 ± 2.94 | 15.99 ± 0.4 | 34.99 ± 22.71 | 17.58 ± 6.05 | 37.42 ± 33.2 | 2.98 ± 4.2 | 360.37 ± 116.42 | 2652.49 ± 633.32 | | |
| + | - | 73 | 8.45 ± 1.39 | 10.41 ± 1.71 | 1.23 ± 0.08 | 7.25 ± 1.03 | 128.49 ± 7.47 | 26.34 ± 3.73 | 15.9 ± 0.44 | 31.8 ± 14.04 | 19.05 ± 13.31 | 39.26 ± 33.8 | 4.27 ± 5.4 | 395.47 ± 121.93 | 2519.2 ± 768.76 | | |
| - | + | 41 | 8.31 ± 1.27 | 10.22 ± 1.52 | 1.23 ± 0.06 | 7.1 ± 0.78 | 129.16 ± 9.27 | 26.54 ± 3.73 | 15.98 ± 0.42 | 32.58 ± 18.02 | 17.33 ± 5.76 | 39.97 ± 33.53 | 3.13 ± 3.89 | 375.8 ± 115.31 | 2790.52 ± 669.94 | | |
| - | - | 35 | 8.3 ± 1.04 | 10.37 ± 1.29 | 1.25 ± 0.1 | 7.44 ± 0.57 | 128.38 ± 6.14 | 26.17 ± 3.13 | 15.86 ± 0.38 | 31.81 ± 11.12 | 18.36 ± 5.7 | 41.19 ± 33 | 4.37 ± 6.69 | 388.39 ± 106.3 | 2603 ± 793.48 | | |
| p-value |  |  | 0.3 | 0.292 | 0.066 | **0.039*** | 0.272 | 0.353 | 0.247 | 0.835 | 0.813 | 0.722 | 0.489 | 0.171 | 0.22 | | |
|  |  |  |  |  |  |  |  |  |  |  |  |  |  |  |  | | |
| *IGF-2(3580) (GG)* | *IGFBP-3 (AA)* |  |  |  |  |  |  |  |  |  |  |  |  |  |  | | |
| + | + | 132 | 8.6 ± 1.08 | 10.64 ± 1.23 | 1.24 ± 0.07 | 7.13 ± 1.63 | 130.2 ± 6.37 | 27.03 ± 3.17 | 15.99 ± 0.41 | 34.49 ± 21.18 | 17.67 ± 5.92 | 39.07 ± 34.82 | 3.09 ± 4.29 | 362.96 ± 116.31 | 2667.21 ± 636.94 | | |
| + | - | 86 | 8.45 ± 1.1 | 10.43 ± 1.3 | 1.24 ± 0.09 | 7.31 ± 0.65 | 128.53 ± 6.2 | 26.25 ± 3.18 | 15.87 ± 0.39 | 32.43 ± 14.08 | 18.31 ± 6.16 | 41.29 ± 36.07 | 4.54 ± 6.08 | 386.4 ± 122.53 | 2562.5 ± 774.84 | | |
| - | + | 24 | 8.32 ± 1.05 | 10.33 ± 1.34 | 1.24 ± 0.07 | 7.23 ± 0.61 | 129.32 ± 6.5 | 26.5 ± 3.17 | 15.93 ± 0.42 | 33.62 ± 23.94 | 16.64 ± 6.25 | 32.71 ± 21.98 | 2.64 ± 2.94 | 372.45 ± 116.13 | 2807.32 ± 682.32 | | |
| - | - | 22 | 8.21 ± 1.86 | 10.27 ± 2.41 | 1.25 ± 0.07 | 7.37 ± 0.66 | 127.29 ± 13.47 | 26.1 ± 4.73 | 15.98 ± 0.36 | 29.37 ± 8.17 | 20.82 ± 22.4 | 34.37 ± 19.46 | 3.38 ± 4.64 | 419.64 ± 87.41 | 2483.23 ± 786.23 | | |
| p-value |  |  | 0.487 | 0.399 | 0.728 | 0.38 | 0.489 | 0.472 | 0.420 | 0.893 | 0.4 | 0.802 | 0.257 | 0.092 | 0.197 | | |
|  |  |  |  |  |  |  |  |  |  |  |  |  |  |  |  | | |
| *IGF-2(3123) (AA)* | *IGFBP-3 (AA)* |  |  |  |  |  |  |  |  |  |  |  |  |  |  | | |
| + | + | 48 | 8.66 ± 1.19 | 10.6 ± 1.41 | 1.23 ± 0.04 | 7.29 ± 0.99 | 130.61 ± 7.03 | 27.26 ± 3.47 | 16.03 ± 0.45 | 36.25 ± 20.19 | 17.94 ± 5.85 | 45.3 ± 36.52 | 2.04 ± 2.33 | 373.14 ± 118.89 | 2599.51 ± 659.21 | | |
| + | - | 29 | 8.46 ± 1.25 | 10.31 ± 1.56 | 1.22 ± 0.03 | 7.24 ± 0.74 | 129.65 ± 6.04 | 26.76 ± 3.04 | 15.94 ± 0.39 | 32.21 ± 11.06 | 17.4 ± 6.47 | 29.95 ± 22.35 | 4.56 ± 5.95 | 383.77 ± 112.58 | 2642.09 ± 755.52 | | |
| - | + | 108 | 8.51 ± 1.02 | 10.58 ± 1.18 | 1.25 ± 0.08 | 7.33 ± 0.6 | 129.41 ± 7.4 | 26.5 ± 3.52 | 15.95 ± 0.47 | 33.52 ± 22.16 | 17.33 ± 6.03 | 34.88 ± 31.25 | 3.46 ± 4.63 | 360.55 ± 114.98 | 2728.44 ± 635.96 | | |
| - | - | 79 | 8.38 ± 1.3 | 10.43 ± 1.59 | 1.25 ± 0.1 | 7.4 ± 0.75 | 128.72 ± 8.72 | 26.39 ± 3.55 | 15.93 ± 0.38 | 31.66 ± 13.86 | 19.35 ± 12.72 | 43.53 ± 36.06 | 4.21 ± 5.8 | 396.63 ± 118.62 | 2511.21 ± 782.64 | | |
| p-value |  |  | 0.504 | 0.759 | 0.434 | 0.427 | 0.494 | 0.503 | 0.583 | 0.664 | 0.529 | 0.07 | 0.246 | 0.167 | | 0.128 |  |

^1^ Z-scores calculated with WHO Child Growth Standards for children up to 5 years old [25] and WHO 2007 reference for children older than 5 years [26].

*p<0.05.

**Table S5-1.** Associations between demographic and pathological features and combinations of IGFBP-3 and two additional genes in the control group.

| **Inhibitor** | **Receptor** | **IGFBP-3** | **n** | **Chronological age, years** | **Bone age, years** | **BA/CA ratio** | **Age of onset, years** | **Z-scores of height^1^** | **Z-scores of weight^1^** | **Z-scores of BMI^1^** | **E2, pmol/l** | **FSH, U/l** | **LH, U/l** | **GH, ng/ml** | **IGF1, ng/ml** | **IGFBP-3, ng/ml** |
| --- | --- | --- | --- | --- | --- | --- | --- | --- | --- | --- | --- | --- | --- | --- | --- | --- |
| *IGF-1(1770) (TT)* | *IGF1R (GG)* | *IGFBP-3 (AA)* |  |  |  |  |  |  |  |  |  |  |  |  |  |  |
| + | + | + | 17 | 8.82 ± 1.73 | 9.12 ± 1.87 | 1.04 ± 0.16 | 8.12 ± 1.73 | 131.36 ± 10.31 | 25.71 ± 2.8 | 16.16 ± 0.93 | 70.37 ± 170.76 | 8.74 ± 7.43 | 4.06 ± 6.37 | 4.17 ± 4.38 | 260.23 ± 117.16 | 1812.82 ± 1495.83 |
| + | + | - | 8 | 8.85 ± 0.77 | 8.38 ± 2.07 | 0.95 ± 0.24 | 8.15 ± 0.77 | 131.42 ± 4.67 | 27.69 ± 2.64 | 16.04 ± 0.36 | 33.91 ± 15.48 | 11.96 ± 5.34 | 4.36 ± 2.71 | 3.51 ± 5.84 | 240.25 ± 60.38 | 2053.16 ± 1098.57 |
| + | - | + | 26 | 8.75 ± 2.06 | 8.65 ± 2 | 1 ± 0.13 | 8.05 ± 2.06 | 130.84 ± 12.03 | 25.56 ± 3.55 | 16.2 ± 1.04 | 29.51 ± 10.22 | 13.63 ± 9.16 | 6.49 ± 10.32 | 3.73 ± 7.56 | 228.86 ± 102.8 | 2386.08 ± 1450.51 |
| + | - | - | 17 | 8.36 ± 1.54 | 8.53 ± 2.62 | 1 ± 0.21 | 7.66 ± 1.54 | 128.55 ± 9.22 | 26.04 ± 3.89 | 15.94 ± 0.43 | 27.95 ± 8.39 | 11.47 ± 8.25 | 4.93 ± 6.11 | 1.85 ± 4 | 259.19 ± 102.63 | 1772.17 ± 1090.51 |
| - | + | + | 46 | 8.69 ± 1.76 | 8.6 ± 2.39 | 0.99 ± 0.18 | 7.86 ± 1.49 | 130.3 ± 10.43 | 25.93 ± 3.52 | 16.1 ± 0.78 | 27.64 ± 10.63 | 9.57 ± 8.83 | 5.03 ± 9.09 | 3.76 ± 6.57 | 258.77 ± 108.44 | 1789.85 ± 1541.6 |
| - | + | - | 37 | 8.77 ± 0.93 | 8.95 ± 1.79 | 1.02 ± 0.16 | 8.07 ± 0.93 | 131.02 ± 5.6 | 27.12 ± 2.58 | 16.03 ± 0.41 | 28.93 ± 9.88 | 8.92 ± 5.65 | 3.86 ± 3.9 | 2.33 ± 4.07 | 275.94 ± 95.35 | 1726.16 ± 1246.42 |
| - | - | + | 43 | 8.8 ± 1.44 | 8.88 ± 1.94 | 1.01 ± 0.16 | 7.99 ± 1.19 | 131.13 ± 8.47 | 26.53 ± 2.61 | 16.1 ± 0.75 | 30.52 ± 12.59 | 10.85 ± 8.83 | 5.87 ± 13.02 | 2.94 ± 3.85 | 232.58 ± 104.64 | 1833.45 ± 1484.47 |
| - | - | - | 31 | 8.89 ± 1.67 | 8.65 ± 2.52 | 0.96 ± 0.19 | 8.13 ± 1.59 | 131.81 ± 10.15 | 25.7 ± 3.13 | 16.18 ± 0.7 | 27.35 ± 12.06 | 7.91 ± 7.29 | 9.06 ± 25.62 | 4.16 ± 4.43 | 293.94 ± 104.51 | 1838.86 ± 1688.46 |
| p-value |  |  |  | 0.996 | 0.993 | 0.875 | 0.996 | 0.996 | 0.457 | 0.995 | 0.807 | 0.227 | 0.369 | 0.064 | 0.109 | 0.806 |
|  |  |  |  |  |  |  |  |  |  |  |  |  |  |  |  |  |
| *IGF-1(6093) (GG)* | *IGF1R (GG)* | *IGFBP-3 (AA)* |  |  |  |  |  |  |  |  |  |  |  |  |  |  |
| + | + | + | 45 | 8.87 ± 1.72 | 8.79 ± 2.21 | 0.99 ± 0.19 | 8.04 ± 1.45 | 131.54 ± 9.79 | 26.04 ± 3.01 | 16.17 ± 0.92 | 44.1 ± 105.37 | 9.23 ± 6.93 | 5.64 ± 9.66 | 3.62 ± 4.38 | 273.33 ± 105.98 | 1915.93 ± 1458.81 |
| + | + | - | 26 | 8.8 ± 0.88 | 8.58 ± 2 | 0.97 ± 0.19 | 8.1 ± 0.88 | 131.14 ± 5.29 | 27.34 ± 2.68 | 16.03 ± 0.39 | 29.91 ± 11.98 | 9.52 ± 5.57 | 4.12 ± 3.54 | 2.83 ± 5.28 | 257.06 ± 82.01 | 1843.4 ± 1169.43 |
| + | - | + | 48 | 8.58 ± 1.75 | 8.69 ± 1.98 | 1.01 ± 0.14 | 7.88 ± 1.75 | 129.85 ± 10.31 | 25.66 ± 3.11 | 16.07 ± 0.85 | 30.74 ± 12.27 | 11.86 ± 9.27 | 5.24 ± 8.16 | 3.31 ± 6.14 | 233.03 ± 107.41 | 2099.74 ± 1479.66 |
| + | - | - | 33 | 8.64 ± 1.73 | 8.82 ± 2.62 | 1 ± 0.19 | 7.88 ± 1.66 | 130.24 ± 10.53 | 26 ± 3.9 | 16.09 ± 0.61 | 27.79 ± 11.27 | 9.54 ± 7.77 | 6.84 ± 17.76 | 2.28 ± 3.37 | 278.36 ± 104.66 | 1803.56 ± 1327.04 |
| - | + | + | 18 | 8.34 ± 1.77 | 8.61 ± 2.45 | 1.02 ± 0.16 | 7.64 ± 1.77 | 128.2 ± 11.49 | 25.49 ± 4.02 | 15.97 ± 0.42 | 26.85 ± 10.57 | 9.63 ± 11.6 | 2.59 ± 3.05 | 4.49 ± 9.08 | 223.76 ± 114.56 | 1496.35 ± 1660.08 |
| - | + | - | 19 | 8.77 ± 0.95 | 9.21 ± 1.55 | 1.05 ± 0.15 | 8.07 ± 0.95 | 131.02 ± 5.68 | 27.07 ± 2.47 | 16.03 ± 0.43 | 29.68 ± 9.91 | 9.39 ± 5.93 | 3.72 ± 3.98 | 2.15 ± 2.82 | 286.75 ± 100.94 | 1703.41 ± 1305.45 |
| - | - | + | 21 | 9.24 ± 1.46 | 9.05 ± 1.91 | 0.98 ± 0.17 | 8.3 ± 0.97 | 133.69 ± 8.43 | 27.41 ± 2.32 | 16.29 ± 0.91 | 28.78 ± 10.37 | 11.99 ± 8.55 | 8.08 ± 18.09 | 3.05 ± 3.79 | 226.96 ± 95.26 | 1908.97 ± 1527.55 |
| - | - | - | 15 | 8.84 ± 1.4 | 8.13 ± 2.36 | 0.91 ± 0.19 | 8.14 ± 1.4 | 131.56 ± 8.47 | 25.43 ± 1.77 | 16.11 ± 0.68 | 27.07 ± 10.02 | 8.37 ± 7.91 | 9.27 ± 27.16 | 5.7 ± 5.47 | 288.83 ± 106.15 | 1840.95 ± 1856.18 |
| p-value |  |  |  | 0.717 | 0.922 | 0.451 | 0.790 | 0.720 | 0.170 | 0.725 | 0.875 | 0.698 | 0.735 | 0.193 | **0.026*** | 0.77 |
|  |  |  |  |  |  |  |  |  |  |  |  |  |  |  |  |  |
| *IGF-2(3580) (GG)* | *IGF2R (GG)* | *IGFBP-3 (AA)* |  |  |  |  |  |  |  |  |  |  |  |  |  |  |
| + | + | + | 66 | 8.46 ± 1.76 | 8.45 ± 2.18 | 1 ± 0.17 | 7.67 ± 1.56 | 129.01 ± 10.35 | 25.59 ± 3.41 | 16.01 ± 0.78 | 28.66 ± 10.77 | 10.82 ± 9.21 | 4.22 ± 6.49 | 3.8 ± 5.83 | 230.97 ± 88.45 | 1862 ± 1556.65 |
| + | + | - | 56 | 8.75 ± 1.26 | 8.63 ± 2.19 | 0.98 ± 0.19 | 8.01 ± 1.2 | 130.89 ± 7.59 | 26.74 ± 2.95 | 16.06 ± 0.51 | 29.07 ± 12.28 | 9.4 ± 7.12 | 5.19 ± 13.57 | 2.66 ± 3.78 | 271.8 ± 95.77 | 1849.96 ± 1364.79 |
| + | - | + | 30 | 8.95 ± 1.64 | 8.73 ± 2.29 | 0.97 ± 0.18 | 8.08 ± 1.33 | 131.99 ± 9.58 | 26.81 ± 3.29 | 16.19 ± 0.82 | 28.32 ± 10.09 | 10.04 ± 9 | 7.11 ± 15.34 | 3.03 ± 3.27 | 259.87 ± 110.44 | 1679.82 ± 1493.32 |
| + | - | - | 19 | 8.37 ± 1.45 | 8.53 ± 2.59 | 1 ± 0.22 | 7.67 ± 1.45 | 128.59 ± 8.76 | 26.11 ± 3.71 | 15.93 ± 0.38 | 28.01 ± 8.47 | 10.1 ± 5.49 | 4.61 ± 3.49 | 2.07 ± 2.87 | 256.03 ± 81.21 | 1836.97 ± 1298.76 |
| - | + | + | 26 | 9.17 ± 1.64 | 9.38 ± 1.68 | 1.03 ± 0.12 | 8.47 ± 1.64 | 133.36 ± 9.6 | 26.35 ± 2.2 | 16.29 ± 0.95 | 57.13 ± 138.17 | 10.9 ± 8.54 | 5.76 ± 10.7 | 3.75 ± 8.23 | 245.92 ± 130.4 | 2297.98 ± 1422.47 |
| - | + | - | 10 | 9.24 ± 1.51 | 9.1 ± 2.33 | 0.98 ± 0.18 | 8.54 ± 1.51 | 133.95 ± 9.12 | 26.93 ± 3.71 | 16.31 ± 0.72 | 29.17 ± 9.72 | 10.01 ± 6.31 | 5.38 ± 7.87 | 3.08 ± 5 | 236.27 ± 61.64 | 2007.78 ± 1291.32 |
| - | - | + | 10 | 9.02 ± 1.62 | 9.4 ± 1.65 | 1.05 ± 0.16 | 8.32 ± 1.62 | 132.57 ± 9.81 | 26.12 ± 2.26 | 16.22 ± 0.93 | 32.07 ± 13.72 | 11.09 ± 6.71 | 8.01 ± 13.15 | 2.8 ± 3.29 | 284.48 ± 139.97 | 2099.32 ± 1337.2 |
| - | - | - | 8 | 8.97 ± 1.15 | 9.38 ± 1.3 | 1.05 ± 0.09 | 8.27 ± 1.15 | 132.24 ± 7.05 | 26.04 ± 0.78 | 16.13 ± 0.6 | 26.8 ± 8.39 | 5.98 ± 7.95 | 13.79 ± 37.67 | 7.01 ± 8.03 | 400.27 ± 99.14 | 1105.88 ± 1578.29 |
| p-value |  |  |  | 0.599 | 0.678 | 0.921 | 0.55 | 0.602 | 0.824 | 0.617 | 0.986 | 0.691 | 0.568 | 0.55 | **0.010*** | 0.626 |
|  |  |  |  |  |  |  |  |  |  |  |  |  |  |  |  |  |
| *IGF-2(3123) (AA)* | *IGF2R (GG)* | *IGFBP-3 (AA)* |  |  |  |  |  |  |  |  |  |  |  |  |  |  |
| + | + | + | 29 | 8.72 ± 1.69 | 8.79 ± 2.14 | 1.01 ± 0.17 | 8.02 ± 1.69 | 130.74 ± 10.06 | 25.73 ± 3.09 | 16.11 ± 0.79 | 31.94 ± 12.48 | 8.98 ± 7.56 | 4.14 ± 5.96 | 4.87 ± 7.51 | 223.14 ± 104.25 | 1853.69 ± 1651.86 |
| + | + | - | 18 | 8.82 ± 1.76 | 8.33 ± 2.87 | 0.93 ± 0.2 | 8.12 ± 1.76 | 131.36 ± 10.69 | 25.85 ± 3.71 | 16.16 ± 0.68 | 31.61 ± 13.2 | 7.51 ± 7.44 | 2.82 ± 3.33 | 2.94 ± 3.55 | 260.98 ± 78.88 | 1885.22 ± 1586.93 |
| + | - | + | 7 | 8.53 ± 2.03 | 9.14 ± 2.85 | 1.04 ± 0.15 | 7.83 ± 2.03 | 129.58 ± 12.09 | 27.26 ± 5.25 | 16.09 ± 0.46 | 23.54 ± 8.65 | 15.65 ± 11.89 | 6.57 ± 5.19 | 2.03 ± 1.4 | 244.56 ± 88.48 | 1717.56 ± 862.27 |
| + | - | - | 6 | 9.16 ± 0.51 | 10.33 ± 1.21 | 1.13 ± 0.09 | 8.46 ± 0.51 | 133.36 ± 2.97 | 28.73 ± 1.68 | 16.17 ± 0.23 | 28.5 ± 6.96 | 9.34 ± 4.31 | 5.94 ± 4.47 | 1.55 ± 1.17 | 280.78 ± 61.67 | 1821.41 ± 1486.93 |
| - | + | + | 63 | 8.63 ± 1.78 | 8.67 ± 2.07 | 1 ± 0.15 | 7.84 ± 1.59 | 130.01 ± 10.45 | 25.8 ± 3.21 | 16.08 ± 0.87 | 38.9 ± 89.32 | 11.7 ± 9.5 | 4.9 ± 8.65 | 3.28 ± 6.05 | 240.75 ± 100.6 | 2045.75 ± 1472.88 |
| - | + | - | 48 | 8.82 ± 1.1 | 8.83 ± 1.91 | 1 ± 0.17 | 8.08 ± 1.03 | 131.35 ± 6.61 | 27.05 ± 2.74 | 16.07 ± 0.5 | 28.12 ± 11.3 | 10.23 ± 6.7 | 6.12 ± 14.84 | 2.64 ± 4.12 | 268.45 ± 97.06 | 1869.61 ± 1261.97 |
| - | - | + | 33 | 9.06 ± 1.54 | 8.85 ± 2.02 | 0.98 ± 0.18 | 8.2 ± 1.25 | 132.68 ± 9.01 | 26.48 ± 2.32 | 16.22 ± 0.9 | 30.47 ± 11.22 | 9.17 ± 7.23 | 7.5 ± 16.03 | 3.17 ± 3.49 | 270.57 ± 122.94 | 1798.94 ± 1557.08 |
| - | - | - | 21 | 8.37 ± 1.5 | 8.33 ± 2.35 | 0.98 ± 0.2 | 7.67 ± 1.5 | 128.62 ± 9.1 | 25.22 ± 3.13 | 15.93 ± 0.49 | 27.4 ± 8.79 | 8.75 ± 7.02 | 7.73 ± 22.92 | 4.11 ± 5.92 | 303.91 ± 118.98 | 1562.9 ± 1404.31 |
| p-value |  |  |  | 0.531 | 0.532 | 0.301 | 0.494 | 0.522 | **0.035*** | 0.540 | 0.376 | 0.365 | 0.335 | 0.760 | 0.131 | 0.909 |

^1^ Z-scores calculated with WHO Child Growth Standards for children up to 5 years old [25] and WHO 2007 reference for children older than 5 years [26].

*p<0.05.

**Table S5-2.** Associations between demographic and pathological features and combinations of IGFBP-3 and two additional genes in the CPP group.

| **Inhibitor** | **Receptor** | **IGFBP-3** | **n** | **Chronological age, years** | **Bone age, years** | **BA/CA ratio** | **Age of onset, years** | **Z-scores of height^1^** | **Z-score of weight^1^** | **Z-scores of BMI^1^** | **E2, pmol/l** | **FSH, U/l** | **LH, U/l** | **GH, ng/ml** | **IGF1, ng/ml** | **IGFBP-3, ng/ml** |
| --- | --- | --- | --- | --- | --- | --- | --- | --- | --- | --- | --- | --- | --- | --- | --- | --- |
| *IGF-1(1770) (TT)* | *IGF1R (GG)* | *IGFBP-3 (AA)* |  |  |  |  |  |  |  |  |  |  |  |  |  |  |
| + | + | + | 21 | 8.49 ± 1.19 | 10.43 ± 1.43 | 1.23 ± 0.05 | 7.34 ± 0.73 | 129.58 ± 7.05 | 26.86 ± 3.63 | 15.95 ± 0.45 | 34.99 ± 16.68 | 18.47 ± 6.04 | 42.06 ± 44.72 | 2.98 ± 3.99 | 330.9 ± 127.41 | 2727.26 ± 782.64 |
| + | + | - | 11 | 8.9 ± 0.73 | 10.73 ± 0.9 | 1.2 ± 0.01 | 7.58 ± 0.29 | 131.95 ± 4.36 | 27.98 ± 2.41 | 16.08 ± 0.32 | 35.73 ± 14.14 | 16.94 ± 5.24 | 38.7 ± 21.15 | 4.18 ± 6.4 | 425 ± 107.34 | 2227.5 ± 297.66 |
| + | - | + | 26 | 8.74 ± 0.92 | 10.69 ± 1.16 | 1.22 ± 0.04 | 7.46 ± 0.64 | 131.01 ± 5.4 | 27.53 ± 2.71 | 16.03 ± 0.32 | 33.31 ± 16.41 | 17.6 ± 5.68 | 42.14 ± 36.33 | 2.8 ± 5.13 | 376.46 ± 133.92 | 2700.97 ± 592.68 |
| + | - | - | 19 | 8.06 ± 2.06 | 9.95 ± 2.53 | 1.24 ± 0.1 | 6.92 ± 1.75 | 126.34 ± 14.72 | 25.76 ± 5.27 | 15.97 ± 0.41 | 33.87 ± 13.14 | 22.44 ± 24.41 | 38.18 ± 40.83 | 2.4 ± 3.46 | 364.8 ± 113.82 | 2529.18 ± 893.56 |
| - | + | + | 47 | 8.47 ± 1.11 | 10.62 ± 1.23 | 1.26 ± 0.08 | 7.36 ± 0.7 | 129.45 ± 6.53 | 26.58 ± 3.16 | 15.94 ± 0.42 | 37.41 ± 28.34 | 16.43 ± 5.52 | 35.45 ± 34.7 | 3.05 ± 3.35 | 355.37 ± 110.44 | 2711.84 ± 674.63 |
| - | + | - | 27 | 8.43 ± 1.16 | 10.44 ± 1.34 | 1.24 ± 0.11 | 7.26 ± 0.59 | 129.23 ± 6.92 | 26.02 ± 3.02 | 15.92 ± 0.49 | 33.22 ± 11.95 | 18.37 ± 5.54 | 50.4 ± 35.26 | 4.4 ± 4.45 | 424.98 ± 99.68 | 2526.1 ± 677.27 |
| - | - | + | 62 | 8.56 ± 1.08 | 10.58 ± 1.26 | 1.24 ± 0.07 | 7.31 ± 0.59 | 129.99 ± 6.44 | 26.91 ± 3.25 | 15.97 ± 0.43 | 32.27 ± 18.97 | 17.98 ± 6.38 | 37.04 ± 26 | 3.11 ± 4.28 | 377.59 ± 107.75 | 2653.12 ± 602.53 |
| - | - | - | 51 | 8.41 ± 1.05 | 10.47 ± 1.35 | 1.25 ± 0.07 | 7.36 ± 0.69 | 129.04 ± 6.18 | 26.52 ± 3.13 | 15.9 ± 0.38 | 29.44 ± 13.41 | 18.12 ± 5.91 | 35.2 ± 31.11 | 4.99 ± 6.91 | 380.04 ± 125.43 | 2632.25 ± 841.76 |
| p-value |  |  |  | 0.732 | 0.984 | 0.476 | 0.512 | 0.729 | 0.545 | 0.769 | 0.392 | 0.843 | 0.162 | 0.401 | 0.115 | 0.521 |
|  |  |  |  |  |  |  |  |  |  |  |  |  |  |  |  |  |
| *IGF-1(6093) (GG)* | *IGF1R (GG)* | *IGFBP-3 (AA)* |  |  |  |  |  |  |  |  |  |  |  |  |  |  |
| + | + | + | 53 | 8.51 ± 1.09 | 10.64 ± 1.18 | 1.25 ± 0.08 | 7.4 ± 0.63 | 129.7 ± 6.45 | 26.73 ± 3.24 | 15.95 ± 0.44 | 37.97 ± 26.92 | 17.45 ± 6 | 36.79 ± 39.03 | 2.94 ± 3.5 | 342.47 ± 114.27 | 2679.53 ± 695.86 |
| + | + | - | 26 | 8.86 ± 0.95 | 10.73 ± 1.08 | 1.21 ± 0.03 | 7.49 ± 0.41 | 131.73 ± 5.71 | 27.37 ± 2.57 | 16.08 ± 0.43 | 34.42 ± 12.66 | 17.02 ± 5.23 | 48.72 ± 34.59 | 4.92 ± 5.44 | 438.29 ± 108.1 | 2421.64 ± 631.93 |
| + | - | + | 62 | 8.76 ± 0.88 | 10.79 ± 1.06 | 1.23 ± 0.06 | 7.48 ± 0.52 | 131.11 ± 5.26 | 27.45 ± 2.64 | 16.03 ± 0.36 | 32.45 ± 18.22 | 17.69 ± 6.15 | 37.95 ± 27.57 | 3.02 ± 4.74 | 375.66 ± 116.98 | 2629.38 ± 579.33 |
| + | - | - | 47 | 8.23 ± 1.55 | 10.23 ± 1.96 | 1.24 ± 0.09 | 7.12 ± 1.23 | 127.74 ± 10.53 | 26.12 ± 4.16 | 15.92 ± 0.4 | 30.35 ± 14.68 | 20.17 ± 16.08 | 34.02 ± 32.54 | 3.91 ± 5.4 | 371.78 ± 123.74 | 2573.16 ± 836.44 |
| - | + | + | 15 | 8.35 ± 1.28 | 10.27 ± 1.62 | 1.23 ± 0.06 | 7.18 ± 0.94 | 128.73 ± 7.49 | 26.45 ± 3.55 | 15.91 ± 0.38 | 32.05 ± 17.98 | 15.67 ± 4.52 | 39.98 ± 34.54 | 3.36 ± 3.73 | 366.69 ± 122.05 | 2847.6 ± 740.67 |
| - | + | - | 12 | 7.94 ± 1.07 | 10.08 ± 1.44 | 1.28 ± 0.15 | 7.04 ± 0.66 | 126.29 ± 6.28 | 25.11 ± 3.23 | 15.73 ± 0.4 | 32.92 ± 12.55 | 19.98 ± 5.51 | 43.31 ± 26.47 | 3.06 ± 3.77 | 396.17 ± 77.95 | 2478.7 ± 563.99 |
| - | - | + | 26 | 8.28 ± 1.29 | 10.19 ± 1.5 | 1.23 ± 0.06 | 7.04 ± 0.7 | 128.36 ± 7.61 | 26.27 ± 3.9 | 15.89 ± 0.48 | 32.89 ± 18.38 | 18.28 ± 6.25 | 39.97 ± 33.62 | 3 ± 4.04 | 381.06 ± 113.37 | 2757.59 ± 638.6 |
| - | - | - | 23 | 8.48 ± 1 | 10.52 ± 1.2 | 1.24 ± 0.06 | 7.48 ± 0.67 | 129.47 ± 5.91 | 26.73 ± 3 | 15.93 ± 0.36 | 31.24 ± 10.56 | 17.51 ± 5.74 | 40.08 ± 36.46 | 5.06 ± 7.78 | 384.33 ± 119.9 | 2667.85 ± 895.08 |
| p-value |  |  |  | 0.199 | 0.614 | 0.195 | 0.02 | 0.164 | 0.28 | 0.199 | 0.631 | 0.653 | 0.094 | 0.641 | 0.077 | 0.56 |
|  |  |  |  |  |  |  |  |  |  |  |  |  |  |  |  |  |
| *IGF-2(3580) (GG)* | *IGF2R (GG)* | *IGFBP-3 (AA)* |  |  |  |  |  |  |  |  |  |  |  |  |  |  |
| + | + | + | 91 | 8.61 ± 1.17 | 10.66 ± 1.33 | 1.24 ± 0.07 | 7.34 ± 0.72 | 130.25 ± 6.9 | 27.04 ± 3.39 | 16 ± 0.44 | 36.15 ± 22.46 | 17.51 ± 5.74 | 38.91 ± 34.76 | 3.25 ± 4.57 | 356.81 ± 116.79 | 2721.34 ± 647.62 |
| + | + | - | 63 | 8.44 ± 1.19 | 10.44 ± 1.41 | 1.24 ± 0.1 | 7.26 ± 0.71 | 129.27 ± 7.05 | 26.45 ± 3.39 | 15.94 ± 0.45 | 31.74 ± 15.26 | 17.42 ± 5.64 | 40.21 ± 35.15 | 4.53 ± 6.01 | 386.92 ± 123.3 | 2512.99 ± 845.69 |
| + | - | + | 41 | 8.59 ± 0.86 | 10.59 ± 1 | 1.23 ± 0.06 | 7.44 ± 0.48 | 130.08 ± 5.09 | 27 ± 2.69 | 15.95 ± 0.34 | 30.82 ± 17.71 | 18.05 ± 6.36 | 39.4 ± 35.38 | 2.74 ± 3.61 | 376.63 ± 115.49 | 2547.08 ± 602.93 |
| + | - | - | 23 | 8.48 ± 0.81 | 10.39 ± 0.94 | 1.23 ± 0.05 | 7.46 ± 0.45 | 129.45 ± 4.81 | 26.65 ± 2.54 | 15.9 ± 0.33 | 34.3 ± 10.21 | 20.77 ± 6.94 | 44.25 ± 39.12 | 4.57 ± 6.39 | 384.98 ± 123.11 | 2698.12 ± 527.11 |
| - | + | + | 16 | 8.36 ± 1.17 | 10.31 ± 1.54 | 1.23 ± 0.07 | 7.18 ± 0.7 | 128.81 ± 6.91 | 26.45 ± 3.5 | 15.9 ± 0.42 | 35.79 ± 28.46 | 17.36 ± 6.98 | 33.21 ± 25.37 | 2.85 ± 2.91 | 370.42 ± 128.42 | 2908.26 ± 681.4 |
| - | + | - | 14 | 7.79 ± 2.14 | 9.93 ± 2.87 | 1.27 ± 0.08 | 6.92 ± 2 | 124.46 ± 15.88 | 24.89 ± 5.24 | 15.9 ± 0.35 | 27.79 ± 7.91 | 23.36 ± 27.95 | 33.03 ± 21.68 | 3.59 ± 4.77 | 416.78 ± 104.46 | 2636.11 ± 902.75 |
| - | - | + | 8 | 8.23 ± 0.82 | 10.38 ± 0.92 | 1.26 ± 0.06 | 7.32 ± 0.41 | 127.96 ± 4.85 | 25.86 ± 2.59 | 15.8 ± 0.34 | 29.29 ± 10.74 | 15.21 ± 4.52 | 31.7 ± 14.36 | 2.23 ± 3.14 | 376.5 ± 94.58 | 2605.45 ± 681.82 |
| - | - | - | 8 | 8.95 ± 0.96 | 10.88 ± 1.25 | 1.21 ± 0.04 | 7.49 ± 0.61 | 132.26 ± 5.63 | 28.23 ± 2.85 | 16.12 ± 0.34 | 32.14 ± 8.38 | 16.38 ± 4.25 | 36.73 ± 15.91 | 3.01 ± 4.7 | 424.63 ± 51.11 | 2215.69 ± 460.89 |
| p-value |  |  |  | 0.396 | 0.657 | 0.34 | 0.75 | 0.444 | 0.436 | 0.609 | 0.254 | 0.456 | 0.967 | 0.67 | 0.407 | 0.137 |
|  |  |  |  |  |  |  |  |  |  |  |  |  |  |  |  |  |
| *IGF-2(3123) (AA)* | *IGF2R (GG)* | *IGFBP-3 (AA)* |  |  |  |  |  |  |  |  |  |  |  |  |  |  |
| + | + | + | 32 | 8.66 ± 1.33 | 10.56 ± 1.56 | 1.22 ± 0.04 | 7.33 ± 0.84 | 130.59 ± 7.87 | 27.23 ± 3.85 | 16.05 ± 0.5 | 37.44 ± 21.87 | 18.97 ± 6.15 | 46.22 ± 36.93 | 1.87 ± 2.05 | 371.13 ± 122.92 | 2633.99 ± 664.59 |
| + | + | - | 21 | 8.42 ± 1.39 | 10.29 ± 1.74 | 1.22 ± 0.04 | 7.16 ± 0.83 | 129.19 ± 8.21 | 26.33 ± 3.83 | 15.96 ± 0.52 | 31.1 ± 11.79 | 16.52 ± 5.7 | 30.76 ± 25.37 | 5.23 ± 6.73 | 390.21 ± 117.03 | 2631.12 ± 805.99 |
| + | - | + | 16 | 8.68 ± 0.87 | 10.69 ± 1.08 | 1.23 ± 0.05 | 7.54 ± 0.51 | 130.66 ± 5.16 | 27.32 ± 2.72 | 15.99 ± 0.35 | 33.88 ± 16.72 | 15.88 ± 4.73 | 43.46 ± 36.8 | 2.39 ± 2.85 | 377.18 ± 114.19 | 2530.54 ± 664.23 |
| + | - | - | 8 | 8.57 ± 0.85 | 10.38 ± 1.06 | 1.21 ± 0.02 | 7.46 ± 0.39 | 130 ± 5.08 | 26.94 ± 2.76 | 15.94 ± 0.36 | 35.14 ± 8.87 | 19.73 ± 8.13 | 27.81 ± 12.37 | 2.79 ± 2.72 | 366.88 ± 105.43 | 2670.88 ± 652.59 |
| - | + | + | 75 | 8.53 ± 1.09 | 10.63 ± 1.27 | 1.25 ± 0.08 | 7.31 ± 0.66 | 129.81 ± 6.46 | 26.84 ± 3.21 | 15.96 ± 0.41 | 35.52 ± 24.01 | 16.85 ± 5.72 | 34.58 ± 31.53 | 3.75 ± 4.94 | 353.6 ± 116.38 | 2798.48 ± 646.07 |
| - | + | - | 56 | 8.29 ± 1.44 | 10.38 ± 1.77 | 1.26 ± 0.11 | 7.22 ± 1.13 | 128.1 ± 9.78 | 26.09 ± 3.84 | 15.92 ± 0.4 | 31 ± 15.17 | 19.24 ± 14.63 | 41.96 ± 35.3 | 4.04 ± 5.43 | 393.16 ± 122.15 | 2499.47 ± 872.33 |
| - | - | + | 33 | 8.45 ± 0.85 | 10.48 ± 0.94 | 1.24 ± 0.07 | 7.37 ± 0.44 | 129.29 ± 5.03 | 26.58 ± 2.67 | 15.9 ± 0.34 | 28.97 ± 16.69 | 18.41 ± 6.63 | 35.57 ± 31.09 | 2.79 ± 3.83 | 376.33 ± 111.89 | 2569.25 ± 591.17 |
| - | - | - | 23 | 8.61 ± 0.88 | 10.57 ± 1.04 | 1.23 ± 0.05 | 7.47 ± 0.52 | 130.24 ± 5.2 | 27.1 ± 2.7 | 15.97 ± 0.34 | 33.26 ± 10.1 | 19.6 ± 6.17 | 47.35 ± 38.41 | 4.64 ± 6.73 | 405.07 ± 111.72 | 2539.8 ± 517.77 |
| p-value |  |  |  | 0.797 | 0.943 | 0.757 | 0.708 | 0.807 | 0.794 | 0.891 | 0.182 | 0.307 | 0.368 | 0.602 | 0.519 | 0.264 |

^1^ Z-scores calculated with WHO Child Growth Standards for children up to 5 years old [25] and WHO 2007 reference for children older than 5 years [26].

*p<0.05.

**Table S6** Summary of the power for given control and CPP groups.

|  | Control | |  | CPP | |
| --- | --- | --- | --- | --- | --- |
| Three combinations of SNPs | IGF-1 (ng/ml) | weight^a^ |  | IGF-1 (ng/ml) | weight^a^ |
| IGF-1(1170)+IGF1R+IGFBP-3 | **0.619** | **0.753** |  | **0.805** | **0.569** |
| IGF-1(6093)+IGF1R+IGFBP-3 | **0.756** | **0.855** |  | **0.642** | **0.588** |
| IGF-2(3580)+IGF2R+IGFBP-3 | **almost 0.999** | **0.309** |  | **0.463** | **0.847** |
| IGF-2(3123)+IGF2R+IGFBP-3 | **0.763** | **0.987** |  | **0.235** | **0.209** |

^a^ z-score transformed
